# Into the fire: Investigating the introduction of cremation to Nordic Bronze Age Denmark: A comparative study between different regions applying strontium isotope analyses and archaeological methods

**DOI:** 10.1371/journal.pone.0249476

**Published:** 2021-05-12

**Authors:** Samantha S. Reiter, Niels Algreen Møller, Bjarne Henning Nielsen, Jens-Henrik Bech, Anne-Louise Haack Olsen, Marie Louise Schjellerup Jørkov, Flemming Kaul, Ulla Mannering, Karin M. Frei

**Affiliations:** 1 Department of Environmental Archaeology and Materials Science, The National Museum of Denmark, Kongens Lyngby (Brede), Denmark; 2 Archaeology Department, Museum Thy, Thisted, Denmark; 3 Vesthimmerlands Museum, Aars, Denmark; 4 Department of Forensic Medicine, Copenhagen University, Copenhagen Ø, Denmark; 5 Department of Ancient Cultures of Denmark and the Mediterranean, The National Museum of Denmark, Copenhagen, Denmark; University at Buffalo - The State University of New York, UNITED STATES

## Abstract

Changes in funerary practices are key to the understanding of social transformations of past societies. Over the course of the Nordic Bronze Age, funerary practices changed from inhumation to cremation. The aim of this study is to shed light on this fundamental change through a cross-examination of archaeometric provenance data and archaeological discussions of the context and layouts of early cremation graves. To this end, we conducted 19 new provenance analyses of strontium isotopes from Early Nordic Bronze age contexts in Thisted County and Zealand and Late Bronze Age contexts from Thisted County and Vesthimmerland (Denmark). These data are subsequently compared with data from other extant relevant studies, including those from Late Bronze Age Fraugde on the Danish island of Fyn. Overall, the variations within our provenience data suggest that the integration and establishment of cremation may not have had a one-to-one relationship with in-migration to Nordic Bronze Age Denmark. Moreover, there seems to be no single blanket scenario which dictated the uptake of cremation as a practice within this part of Southern Scandinavia. By addressing *habitus* in relation to the deposition of cremations as juxtaposed with these provenance data*¸* we hypothesize several potential pathways for the uptake of cremation as a new cultural practice within the Danish Nordic Bronze Age and suggest that this may have been a highly individual process, whose tempo may have been dictated by the specificities of the region(s) concerned.

## Introduction

During the Early to Late Nordic Bronze Age transition (c. 1100 BC), funeral practices in Southern Scandinavia exhibited a gradual but very marked changeover. Rather than continuing the extant tradition of inhuming their dead, Nordic Bronze Age peoples began to near exclusively cremate them instead [1]. In fact, cremation was so strongly associated with Late Bronze Age contexts in Europe, that one of the dominant cultures of the time (the Urnfield Culture) takes its name from the fields of urns in which the cremated dead were placed [2]. While scholars have recorded various examples of cremation in Scandinavia from the Mesolithic and continuing through to the 10^th^ c. AD [3], the depositional practices which characterize those first scattered instances of more intensified cremation present a fascinating variety of compositions, especially when juxtaposed with the more uniform urn burials which characterize later periods. Very broadly, cremations in the Early Nordic Bronze Age (a period of time equivalent to the Central European Middle Bronze Age) included single individuals and multiple individuals buried in single or multiple contexts, which sometimes exclusively contained cremated material or, on occasion, combinations of both inhumed and cremated remains within the same deposition [4–10]. By contrast, Late Bronze Age cremations were generally deposited inside specific cremation urns.

Research suggests that cultural exchange (such as indicated e.g. by the arrival of new funeral practices) is an interface between the circulation of objects, ideas and people [11, 12]. It is also clear that, in pre-literate societies such as those in the Nordic Bronze Age, ideas cannot have travelled without the active involvement of human carriers [13]. While the Nordic Bronze Age region was late to adopt cremation when compared with the rest of Europe, archaeological evidence suggests that the practice did not arrive in Scandinavia in a wave-like fashion (i.e. one which advanced from Southern Scandinavia to points north). There were some areas (such as e.g. Thisted County in northwest Jutland) which exhibited a very early emergence of cremation and in which that funerary practice was practiced comparatively more frequently (in Thisted County, cremation counts for ca. 10% of the total Period II (1500–1300 BC) burials recorded to date [14]).

Given the mounting evidence underscoring Neolithic and Bronze Age mobility throughout Europe [15–27], it seems tempting to imagine a scenario in which the *avant garde* trendsetters who introduced cremation would have been non-locals. (Within this context, we consider locals to be persons whose ^87^Sr/^86^Sr lies within the strontium isotope baseline range for present-day Denmark of 0.7081–0.71111. Non-locals, by extension, would exhibit ^87^Sr/^86^Sr either above or below this range.) The introduction of change by dint of immigration is also in line with one of the more popular explanations for changes in burial practice: population changeover [8, 28, 29]. However, recent discussions of archaeological cremation contexts suggest that the social processes surrounding the organization of cremations in general [30] as well as the treatment and deposition of the first cremated bodies in the Early Bronze Age in particular [7, 8, 10, 31] may be more in line with cultural continuity than demographic change.

This study aims to shed light on the introduction and eventual entrenchment of cremation as a burial practice in Denmark. To this end, we query whether the cultural adoption of cremation and its eventual establishment as the dominant mode of funerary deposition coincided with noticeable patterns of immigration. Our method for determining immigration is through strontium isotope analysis of cremated material and inhumed material in direct association with cremated material. In order to gain a handle on immigration over time, we have concentrated specifically on Early Nordic Bronze Age cremation contexts (i.e. before the onset of cremation as a standardized form of funerary deposition) as opposed to Late Nordic Bronze Age contexts (i.e. in which the deposition of cremations had become more standardized). Therefore, this manuscript presents new provenance data from strontium isotope analyses conducted on human and animal material from three Danish study areas: Early Nordic Bronze Age material from Thisted County (Jutland) and the island of Zealand and Late Bronze Age material from Thisted County and the region of Vesthimmerland (both from Jutland). These data are then compared with the results of previous investigations of Late Nordic Bronze Age/Early Iron Age cremations from Viborg (Jutland) and Fraugde Parish on the Danish island of Fyn [32].

Here, we present 19 strontium isotope analyses from ten sites (Villerup, Egshvile, Erslev, Nørhågård and Ginnerup from Thisted County, Hvidegaard and Maglehøj from Zealand and from three different (but closely-situated) sites in the Stenildgård area in Vesthimmerland). In selecting our samples, we were careful to choose graves which could furnish human skeletal tissues less susceptible to environmental contamination [33]. Unfortunately, this (as well as the preservation of material and its selection for inclusion within cremation graves in antiquity) somewhat limits the array of graves suitable for analysis. Nonetheless, our present data include provenance indicators for fifteen people from Early Nordic Bronze Age Periods II-III (1500–1100 BC) and Late Nordic Bronze Age Periods IV-V (1100–800 BC). Of the 19 analyses, one (Erslev NM 175/29, Grave C, Period II) was of an inhumation interred concurrently with a cremation; all others represented cremated material. In addition, we include the results of provenance analyses conducted on two examples of burned faunal material recovered together with cremated human remains: a cremated *ovid/capra* found associated with the cremation buried with the inhumation at Nørhågaard (THY 1550 Grave N3, cremated material from niche) and a cremated boar’s tusk from Stenildgård (found together with the Late Bronze Age Urn A22 from VMÅ 2560 x 133).

In our discussion of the results, we compare data with other published work in order to gain perspective on the potential currents of cultural change within the dynamic world of the European Bronze Age [34–40]. When placed in relation to considerations of context and the varied layouts of the Early Bronze Age cremations as opposed to Late Bronze Age cremations, we hypothesize four possible scenarios through which cremation may have been first taken up and then entrenched within our study regions. Finally, we reflect on the new questions which arise from this study and suggest potential avenues for future research.

## Description of the sites

In order to be able to accurately compare and contrast the different depositional constellations of the cremations and the single combined cremations and inhumation which were analyzed from the three study areas, we provide generalized descriptions of the sampled funeral contexts by period (i.e. Early or Late Nordic Bronze Age) and region. See below. For more detailed descriptions of the sites and the specific find contexts of each of our samples, please see S1 Appendix. For ease of comparison relating to the periods over which the various samples were deposited, we direct the reader to Table 1.

**Table 1:** Summary of chronological timespans associated with Periods II-V of the Nordic Bronze Age.

Category	Period	Duration
Early Nordic Bronze Age	Period II	1500–1300 BC
	Period III	1300–1100 BC
Late Nordic Bronze Age	Period IV	1100–950 BC
	Period V	950–800 BC

This data is drawn from the results of recent ^14^C data from cremated bone (after [41]).

### Early Bronze Age material from Thisted County

**Fig 1:**
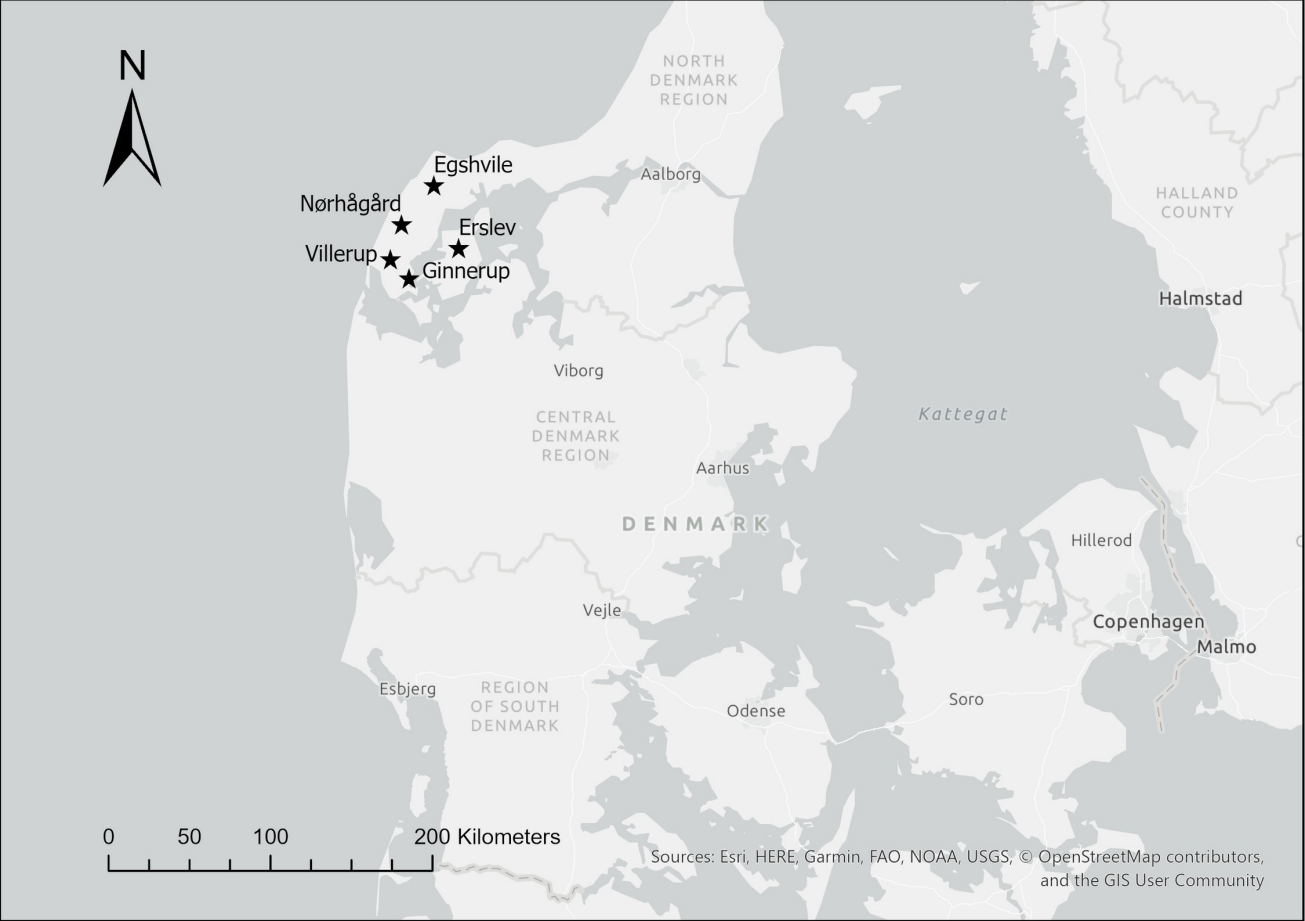
Map showing locations of the Early Nordic Bronze Age sites analyzed within this paper from Thisted County, Denmark. This map was generated using ESRI ArgGIS Pro software and basemaps, licensed to the National Museum of Denmark. The base map and data are from OpenStreetMap and OpenStreetMap Foundation.

This study conducted strontium isotope analysis on the remains of seven human individuals and one *ovid/capra* from five burial mound sites within Thisted County: Villerup, Egshvile, Erslev, Nørhågård and Ginnerup. See Fig 1. Overall, our material included Early Bronze age cremations with the following variations: a single cremated subadult in one pile of remains within a central cist (Villerup THY1696, Grave N106 and Egshvile THY2554, Urn N6 x 18), two cremated subadults within a single textile-wrapped context in a central cist (Ginnerup THY5055, N16), a single cremated individual divided into two separate piles of remains (Villerup THY1696, Grave N10), an inhumed individual in a central grave with one unburnt cranium and a cremation near the inhumation’s feet (Erslev NM 175/29, Grave C) and an inhumation placed in an oak coffin at the foot of which lay an offset niche containing both cremated human and *ovid/capra* remains (Nørhågård THY1550, N3). See Table 2 for sample specifications.

**Table 2:** Details of material sampled from Early Nordic Bronze Age Thisted County.

Site	Grave	Type	Period	Sex	Age	What analyzed	Sample No.
Villerup	THY 1696 grave N106	Cremation	Period II	?	7–13 mo.	dm** (enamel)	KF2050
Villerup	THY 1696 grave N10	Cremation	Early Nordic Bronze Age	♀?	**?**	PM1** (enamel)	KF2051
Egshvile	THY 2554 urn N6	Cremation	Period II/ AAR-8826 3052 +/-46 BP* [42]	?	5 yr.	M1** (enamel)	KF2052
Erslev	NM 175/29 grave C	Combined cremation & inhumation	Period II	?	?	M2** (enamel)	KF2049
Nørhågård	THY 1550 grave N3 (cremated material from niche-*H*. *sapiens*)	Combined cremation & inhumation incl. cremated faunal material	Inhumation associated with this cremation Period III/ OxA-28991 2949 +/- 28* & OxA-28992 2943 +/-28* [17]	♀?	Adult	*pars petrosa*	KM213
Nørhågård	THY 1550 grave N3 (cremated material from niche, faunal-*ovid/capra*)	Combined cremation & inhumation incl. cremated faunal material	Inhumation associated with this cremated faunal material Period III/ OxA-28991 2949 +/- 28* & OxA-28992 2943 +/-28 [17]	N/A	N/A	molar enamel	KM127
Ginnerup	THY 5055, grave N16 (4 yr. old)	Cremation	Early Nordic Bronze Age/ AAR20592 2966 +/-25* AAR20596 2910 +/-25* [5,4]	?	4 yr.	lower dm1 (enamel)	KF1835
Ginnerup	THY 5055, grave N16 (4 yr. old)	Cremation	Early Nordic Bronze Age/ AAR-20592 2966 +/-25* AAR-20596 2910 +/-25* [5,4]	?	4 yr.	upper di1 (enamel)	KF1836

The light grey shading denotes samples of different tissues likely from the same individual. The dark grey shading denotes samples from cremated faunal material found in association with cremated human material.

*NB: All ^14^C dates are listed at the 1-sigma confidence level.

**NB: Because of the explosive effects of the high temperatures of the cremation pyres on dental enamel, it was not always possible to identify the teeth more specifically.

### Early Bronze Age material from Zealand

**Fig 2:**
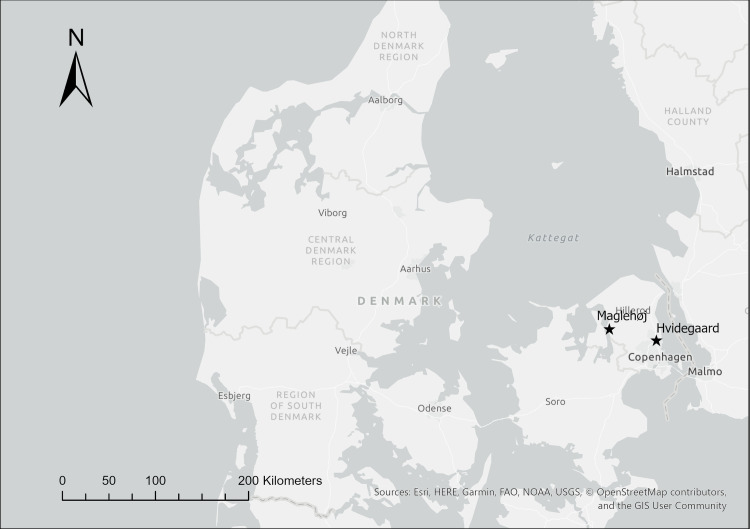
Map showing locations of the Early Nordic Bronze Age sites analyzed within this paper from Zealand, Denmark. This map was generated using ESRI ArgGIS Pro software and basemaps, licensed to the National Museum of Denmark. The base map and data are from OpenStreetMap and OpenStreetMap Foundation.

Two Early Bronze Age Period III (1300–1100 BCE) sites from Zealand were also included in the present study: Hvidegaard and Maglehøj. See Fig 2 and Table 3. While the ritually-charged contexts of these two sites merit further discussion at a future date, the further variations in the depositional practices relating to cremation exhibited by Hvidegaard and Maglehøj offer an excellent counterpoint to the sites presented thus far. The grave from Hvidegaard (NM B 9220) was comprised of e.g. a large pile of cremated remains wrapped in a wool cloth and placed on a cowhide surrounded by unburned artefacts (including a sort of ritual bag containing various unburnt animal bones and natural objects, a so-called “sorcerer’s bag” [43]. The configuration of the grave was such that it presented the wrapped cremated remains as if they comprised a single inhumed body. Similarly, the grave from Maglehøj (NM B 4092–95) also included a pile of cremated remains wrapped in a wool cloth. A belt box associated with this cremation included, once again, various different unburnt animal remains as well as some small cremated bones of human origin [44].

**Table 3:** Details of material sampled from Early Nordic Bronze Age Zealand.

Site	Grave	Type	Period	Sex	Age	What analyzed	Sample No.
Hvidegaard	NM B 9220	Cremation	III	♂?	18–25 yrs (young adult)	*pars petrosa*	KM212
Maglehøj	NM B 4092–95	Cremation	III	♀?	≤ 30 yr?	*pars petrosa*	KM210

### Late Bronze Age material from Thisted County

**Fig 3:**
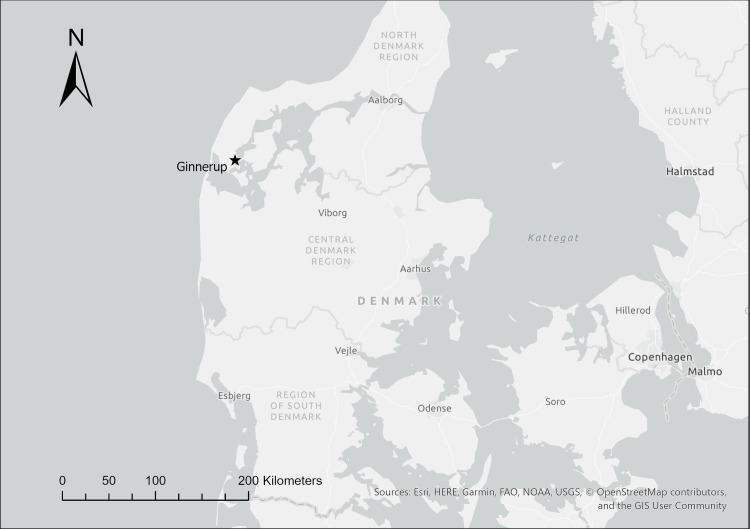
Map showing location of the Late Bronze Age site analyzed within this paper from Thisted County, Denmark. This map was generated using ESRI ArgGIS Pro software and basemaps, licensed to the National Museum of Denmark. The base map and data are from OpenStreetMap and OpenStreetMap Foundation.

One Late Bronze Age cremation grave from Thisted was included as a point of comparison. The sample came from a site from which we also sampled Early Bronze Age material (Ginnerup THY 5055). See Fig 3 and Table 4. The Late Bronze Age cremation analyzed from this site involved cremated remains placed in a small wooden cist within the same mound from which the Early Bronze Age materials described above were also sampled (Ginnerup THY5055, N16).

**Table 4:** Details of material sampled from Late Nordic Bronze Age Thisted County.

Site	Grave	Type	Period	Sex	Age	What analyzed	Sample No.
Ginnerup	THY 5055, grave N30	Cremation	IV	♂?	?	*pars petrosa*	KM211

### Late Bronze Age material from Stenildgård, Vesthimmerland

**Fig 4:**
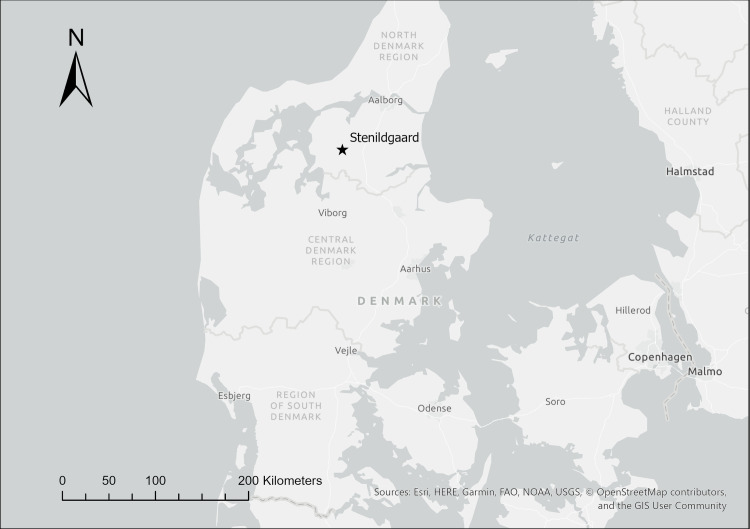
Map showing location of the Stenildgård area. The sites described below are all within <2 km distance of each other. This map was generated using ESRI ArgGIS Pro software and basemaps, licensed to the National Museum of Denmark. The base map and data are from OpenStreetMap and OpenStreetMap Foundation.

While all the contexts sampled for the present research from the Stenildgård region are Late Bronze Age, they nonetheless come from an area in which early cremations have already been documented and investigated in terms of provenance [15]. In addition to Single Grave Culture and Dagger Period contexts, the region also includes a series of Late Neolithic/Bronze Age mounds, which are the primary interest of the current research. Originally thought to have numbered approximately 20, the large group of mounds in this part of Aars, Vesthimmerland has been collectively known since the 1800s as the “Galgehøje” (lit. “gallows hills”). Over the last two decades, several archaeological rescue interventions were necessitated in the advance of housing developers, which were the source of the material analyzed here. All of the Late Bronze Age samples from this study area were taken from urns in cists placed in association with mounds. See Fig 4 and Table 5. All finds could be dated to the Late Bronze Age. In their placement at the periphery of the Late Neolithic/Early Bronze Age mounds in small, often stone-lined cists, these urns follow the typical schema for the deposition of cremations which came to characterize the later part of the Bronze Age [45] and which can be contrasted with the marked variation of earlier periods [46–48].

**Table 5:** Details of material sampled from Late Nordic Bronze Age Stenildgård, Vesthimmerland.

Site	Grave	Type	Period	Sex	Age	What analyzed	Sample No.
Stenildgård	VMÅ 2883 x 174 Urn A521	Cremation	Late Nordic Bronze Age	?	?	*pars petrosa*	KF1837
Stenildgård	VMÅ 2883 x 136 Urn A267	Cremation	Late Nordic Bronze Age	?	?	lower first or second premolar*	KF1838
						(enamel)	
Stenildgård	VMÅ 2883 x 202 Urn A349	Cremation	Late Nordic Bronze Age	?	?	*pars petrosa*	KF1839
Stenildgård	VMÅ 2560 x 28 Urn A23	Cremation	Late Nordic Bronze Age	♂?	?	*pars petrosa*	KF1840
Stenildgård	VMÅ 2542 x 13 Urn A4	Cremation	Late Nordic Bronze Age	?	?	lower M2* (enamel)	KF1841
Stenildgård	VMÅ 2560 x 132 Urn A22	Cremation, incl. cremated faunal material	Late Nordic Bronze Age	♂?	Adult	*pars petrosa*	KF1842
Stenildgård	VMÅ 2560 x 133 Urn A22	Cremation, incl. cremated faunal material	Late Nordic Bronze Age	N/A	N/A	boar tusk (enamel)	KF1843
Stenildgård	VMÅ 2560 x 133 Urn A22	Cremation, incl. cremated faunal material	Late Nordic Bronze Age	♂?	Adult	upper M2* (enamel)	KF1844

The light grey shading denotes samples of different tissues possibly from the same individual. The dark grey shading denotes samples from cremated faunal material found in association with cremated human material.

* NB: Because of the explosive effects of the high temperatures of the cremation pyres on dental enamel, it was not always possible to identify the teeth more specifically.

## Material and methods

This study analyzed human skeletal tissue from prehistoric individuals. Sampling permissions were obtained from Museum Thy, Vesthimmerlands Museum and the National Museum of Denmark, all three of which curate the collections concerned. As strontium isotope analyses were the main focus of this paper, we have detailed the methods utilized therein below. In order to facilitate cross-comparison of osteological observations (especially MNI) between the different sites and study areas, the remains from the two cremations from Zealand (Hvidegaard and Maglehøj) underwent osteological assessment by Jørkov. The methods utilized for those analyses can be found in S2 Appendix.

### Strontium isotope analyses

Strontium isotope analyses conducted on the tooth enamel of archaeological human remains can provide information on provenance and potential mobility at the individual level [49, 50]. Recent studies have also suggested that the cremation process does not affect the strontium isotope composition of the otic capsule in the petrous bone or of tooth enamel [32, 51, 52], thereby providing a tremendous resource for information about mobility. Given the heterogeneous material available, this study included two types of tissues: the petrous bone and tooth enamel. Both tissue types were tested from cremated individuals. Our dataset also includes the results of the analyses of the dental enamel from one inhumed person. See Tables 2–5 above.

It is important to mention that our ^87^Sr/^86^Sr data do not represent the same periods of the human life span. As we are limited by the depredations of time, in practical terms, the material available for study determines the life period(s) that can be studied by conducting strontium isotope analysis on a particular individual. For example, the mineralization of the inner periosteal layer of the *pars petrosa* occurs *in utero* and undergoes no further remodelling after the age of two years [53–56]. Therefore, analyses of *pars petrosa* samples allow for a glimpse into the region from a person was nourished from the womb up until age two (based on an average of food consumption). Likewise, the mineralization of tooth enamel occurs within different times over the life course from childhood to early adolescence (i.e. the formation of the first premolar takes place from ca. 18 months to ca. 6 years, the second premolar from two to seven years, the first molar’s tooth enamel takes place *in utero* until ca. 3 years of age, the second molar between the ages of ca. 2–8 years and the third molar from ca. 7–16 years) [57–59]. In our study, we also analyzed several deciduous teeth. Studies have shown that deciduous molars (such as those tested from the Villerup (THY 1696 grave N106) and Ginnerup (THY 5055 grave N16) mineralize between 15 weeks *in utero* to the age of 11 months [60]. Upper deciduous first incisors, such as that tested from Ginnerup (THY 5055 grave N16) mineralize between 14 weeks *in utero* to the age of 5.5 months [60].

Tooth enamel samples from both inhumations and cremations were pre-cleaned by removing the enamel’s surface with a drill bit, and subsequetly, a few milligrams of enamel were sampled from each tooth. In accordance with the procedures outlined by Harvig et al. [32], we sampled the otic capsules of *pars petrosae* from individuals from Ginnerup (THY 5055 grave N30), Nørhågård (THY 1550, grave N3 cremation), Hvidegaard (NM B 9220) and Maglehøj (NM B 4092–95) as well as from the following graves from the Stenildgård region (VMÅ2883 x 174 Urn A521, VMÅ 2883 x202 Urn A349, VMÅ 2560 x28 Urn A23 and VMÅ 2560 x132 Urn A22). *Pars petrosa* samples were pre-cleaned by removing the surface of the bone with a drill bit after extant methodologies [32, 61]. Subsequently, a few milligrams of bone were drilled out directly from the otic capsules and collected for further processing.

The tooth enamel and powdered bone samples were dissolved in precleaned 7 ml Teflon beakers (Savillex) in a 1:1 solution of 0.5 ml 6 N HCl (Seastar) and 0.5 ml 30% H_2_O_2_ (Seastar). The samples typically dissolved within five minutes, after which the solutions were dried on a hotplate at 80°C. Subsequently, the enamel samples were taken up in a few drops of 3N HNO_3_ and then loaded onto disposable 100 μl pipette tip extraction columns into which we fitted a frit which retained a 0.2 ml stem volume of intensively pre-cleaned mesh 50–100 SrSpec (TrisKem) chromatographic resin. The elution recipe essentially followed that by Horwitz et al. [62], albeit scaled to our needs insofar as strontium was eluted / stripped by pure deionized water and then the eluate dried on a hotplate.

Thermal ionization mass spectrometry was used to determine the Sr isotope ratios. Samples were dissolved in 2.5 μl of a Ta_2_O_5_-H_3_PO_4_-HF activator solution and directly loaded onto previously outgassed 99.98% single rhenium filaments. Samples were measured at 1250–1300°C in a dynamic multi-collection mode on a VG Sector 54 TI mass spectrometer equipped with eight Faraday detectors (Institute of Geosciences and Natural Resource Management, University of Copenhagen). Five nanogram loads of the NIST SRM 987 Sr standard that were ran during the time of the project yielded ^87^Sr/^86^Sr = +/- (n = XX5, 2σ), which we compare to the generally accepted value of ^87^Sr/^86^Sr = 0.710248.

## Results

Overall, the results of the strontium isotope analyses conducted on the study material from all three regions ranged from ^87^Sr/^86^Sr = 0.70989–0.714090. The ranges obtained from the individuals from Thisted County range between ^87^Sr/^86^Sr = 0.70989 to 0.71205. The cremated remains from Zealand were ^87^Sr/^86^Sr = 0.71193 and ^87^Sr/^86^Sr = 0.71095 for Hvidegaard and Maglehøj, respectively. Finally, the strontium isotope analysis conducted on the Late Bronze Age contexts from Vesthimmerland ranged between ^87^Sr/^86^Sr = 0.710608–0.714090. See Table 6. In order to interpret these results, it is necessary to have knowledge of the local bioavailable strontium isotope baseline range. While different kind of proxy materials have been used for the purpose of establishing bioavailable baseline ranges of specific regions, scholars have yet to reach a consensus regarding which type of proxy (e.g. surface water, plants, soil, fauna, etc.) is the most suitable means of delimiting the strontium isotope baseline range for a specific area [63]. Several baselines have been established for Denmark based on different types of environmental samples including surface waters, plants, soil leachates and fauna [20, 64–69]. Furthermore, a recently published baseline study from almost 1200 soil samples taken throughout Europe adds yet another layer of data [70]. Combined with recent work regarding the Danish water system [71], these studies seem to indicate that the local bioavailable baseline for the area of present-day Denmark (excluding the island of Bornholm) is ^87^Sr/^86^Sr = 0.7081 to 0.7111.

**Table 6:** Results of strontium isotope analyses of Early Nordic Bronze Age material from Thisted County and Zealand and Late Nordic Bronze Age material from Thisted County and Vesthimmerland.

Site Name	Grave/individual	What analyzed	Sample No.	^87^Sr/^86^Sr	(±2SE)
**Early Nordic Bronze Age Thisted County**					
Villerup	THY 1696, Grave N106	dm (enamel)	KF2050	0.71099	0.00001
Villerup	THY 1696, Grave N10	PM1 (enamel)	KF2051	0.71082	0.00001
Egshvile	THY 2554, Urn N6	M1 (enamel)	KF2052	0.71205	0.00001
Erslev	NM 175/29, Grave C	M2 (enamel)	KF2049	0.71025	0.00001
Nørhågård	THY 1550, Grave N3 (niche with cremated material)	*pars petrosa*	KM213	0.71040	0.00001
Nørhågård	THY 1550, Grave N3 (niche with cremated material)	ovid/capramolar (enamel)	KM127	0.71041	0.00001
Ginnerup	THY 5055, Grave N16 (4 yr. old)	lower dm1 (enamel)	KF1835	0.71019	0.00001
Ginnerup	THY 5055, Grave N16 (4 yr. old)	upper di1 (enamel)	KF1836	0.70989	0.00001
**Early Nordic Bronze Age Zealand**					
Hvidegaard	NM B 9220	*pars petrosa*	KM212	0.71193	0.00001
Maglehøj	NM B 4092–95	*pars petrosa*	KM210	0.71095	0.00001
**Late Nordic Bronze Age Thisted County**					
Ginnerup	THY 5055, grave N30	*pars petrosa*	KM211	0.71009	0.00002
**Late Nordic Bronze Age Vesthimmerland**					
Stenildgård	VMÅ 2883 x 174 Urn A521	*pars petrosa*	KF1837	0.712108	0.00001
Stenildgård	VMÅ 2883 x 136 Urn A267	lower PM1 or PM2 (enamel)	KF1838	0.712030	0.00001
Stenildgård	VMÅ 2883 x 202 Urn A349	*pars petrosa*	KF1839	0.710608	0.00001
Stenildgård	VMÅ 2560 x 28 Urn A23	*pars petrosa*	KF1840	0.711115	0.00001
Stenildgård	VMÅ 2542 x 13 Urn A4	lower M2 (enamel)	KF1841	0.712001	0.00001
Stenildgård	VMÅ 2560 x 132 Urn A22 with cremated *H*.*sapiens* and faunal *sus*	*pars petrosa*	KF1842	0.711516	0.00001
Stenildgård	VMÅ 2560 x 133 Urn A22 with cremated *H*.*sapiens* and faunal *sus*	boar tusk (enamel)	KF1843	0.714090	0.00001
Stenildgård	VMÅ 2560 x 133 Urn A22 with cremated *H*.*sapiens* and faunal *sus*	upper M2 (enamel)	KF1844	0.711768	0.00001

The light grey shading denotes samples which are likely to come from the same individuals. The dark grey shading denotes samples from cremated faunal material found in association with cremated human material.

### Results Early Nordic Bronze Age Thisted County

In the Thisted County, our results reveal the presence of individuals whose isotopic values fall within the local baseline range as well as an individual whose values fall outside the local bioavailable baseline range. In this study region, the six Early Bronze Age individuals that yielded local values range from ^87^Sr/^86^Sr = 0.70989 to 0.71099. These include the individuals from the sites of Villerup (THY1969 graves N106 and N10), Erslev (NM 175/29 grave C), Nørhågård (THY 1550 grave N3 cremated human remains from niche) and Ginnerup (THY 5055 grave N16, from which we have two samples from the same individual). In addition, the *ovid/capra* faunal sample from Nørhågård (THY 1550 grave N3, cremated material from niche) also yielded a strontium isotope ratio within the range of what is considered local for present-day Denmark. The only individual from Thisted County that yielded a strontium isotopic value outside the local baseline range was the cremated subadult individual from Egshvile (THY 2554 urn N6), whose ^87^Sr/^86^Sr = 0.71205.

### Early Nordic Bronze Age Zealand

From the samples from Zealand our results from the two cremation samples yielded both local and non-local isotopic values. Maglehøj yielded ^87^Sr/^86^Sr = 0.71095, which falls within the local baseline range for Zealand and mainland Denmark, while Hvidegaard yielded ^87^Sr/^86^Sr = 0.71193 and thereby falls outside the local baseline range.

As previous osteological analyses by Arcini as referred to by Goldhahn [48] identified several individuals in the Hvidegaard burial, we conducted additional physical anthropological analyses in the present study. See S2 Appendix. Our present analyses of Grave NM B 9220 (known as the Hvidegaard shaman grave) suggest the presence of the cremated remains of a minimum of two individuals; a young adult (18–25 years), who showed some signs of male dimorphism and a young subadult (1–2 years). See S2 Appendix. This can be compared with the results of our analyses of the Maglehøj burial (NM B 4092–95) which suggests the presence of a single individual, likely 30 years or older, who demonstrated signs of female dimorphism. See S2 Appendix.

### Results Late Nordic Bronze Age Thisted County

Our single Late Bronze Age urn cremation from Thisted County revealed ^87^Sr/^86^Sr = 0.71009, which falls within the local baseline for present-day Denmark.

### Results Late Nordic Bronze Age Vesthimmerland

From the Stenildgård region in Vesthimmerland, our strontium isotope analyses reveal a range from ^87^Sr/^86^Sr = 0.710608 to 0.712108. The single faunal sample from the boar’s tusk (KF 1843) demonstrated ^87^Sr/^86^Sr = 0.714090. Of the total of seven individuals investigated, all but two individuals yielded strontium isotopic values that fall outside the local baseline for Denmark. The individuals that yielded non-local values range from ^87^Sr/^86^Sr = 0.711516 to 0.712108, and are the individuals from VMÅ 2883 Urn A521, VMÅ 2883 Urn A267, VMÅ 2560 Urn A22 (*pars petrosa* and second molar, both possibly from the same individual) and VMÅ 2542 Urn A4.

### Overview of results

Overall, with the exception of Egshvile (THY 2554, Urn N6) and Hvidegaard (NM B 9220), our results reveal that the majority of the Early Bronze Age material sampled from Thisted County and Zealand exhibited ^87^Sr/^86^Sr values within the range which would be considered local to present-day Denmark. This contrasts with the Late Bronze Age data from the Stenildgård sites in Vesthimmerland in which the Late Bronze Age material analyzed seems to mostly represent non-locals. Nevertheless, a single datapoint from Thisted (Ginnerup THY 5055 N30) represents an Early Nordic Bronze Age cremation outside the local range for present day Denmark, and two datapoints from Stenildgård (VMÅ 2883, Urn A349 and VMÅ 2560 Urn A23) represent Late Bronze Age cremations which lie within the local range for present-day Denmark.

## Discussion

During the last decade, several mobility studies based on strontium isotope analyses of human remains from inhumations have been conducted on (or including) the area of present-day Denmark [15–17, 20, 27, 64–68, 70–74]. A relatively large number of them focus on (or include) human remains from various periods within the Nordic Bronze Age [16, 17, 20, 27, 72, 73, 75], thereby providing a large body of background data against which to compare the results of the present investigations. Recent advances in method development for the analysis of strontium isotopes of cremated remains [32, 61, 76] open up new opportunities to investigate mobility and social dynamics in the prehistoric periods in which cremation was the norm, like e.g. the Late Nordic Bronze Age. Additionally, it provides the possibility to shed light on the introduction of this new practice, thereby opening up new avenues of discussion on the topic. In order to address our main research objective (i.e. examining the uptake and establishment of cremation in the Nordic Bronze Age), it is first upon the earlier cremations which we will focus our debate; discussion of the Late Nordic Bronze Age results will follow thereafter.

### Early Nordic Bronze Age data

As previously mentioned, Thisted County was one of the regions within the Nordic Bronze Age region which exhibited an early uptake of cremation. Two of the other Bronze Age regions in Northern Europe which also exhibited an early adoption of cremation are the Frisian Islands [14] and the island of Bornholm [77, 78]. Importantly, in relation to the present study, both of these areas also have ^87^Sr/^86^Sr baseline ranges which partially overlap the range of present-day Denmark [79, 80]. Therefore, from the basis of the strontium isotope data examined here, we cannot exclude in-migration to Denmark from parts of Europe in which cremation was common at this time, such as is the case with the e.g. the Frisian Islands and Bornholm or with other regions with ^87^Sr/^86^Sr baselines which overlap with that of present-day Denmark.

In the following, we will have a closer look at the individuals that exhibit values which are outside of this range, suggesting that they are non-locals. The subadult (estimated to be a five-year-old) buried in an urn within the central grave at the mound at Egshvile (THY 2554, Urn N6) yielded a ^87^Sr/^86^Sr = 0.71205. The other non-local came from the composite deposition of two individuals (one adult, one subadult) at Hvidegaard (NM B 9220) of which the adult male’s ^87^Sr/^86^Sr = 0.71193. Both the graves from Egshvile (THY 2554, Urn N6) and Hvidegaard (NMB 9220) (Periods II and III, respectively) yielded Sr isotope ratios that we interpret as non-local values. In addition to being examples of comparatively early funeral depositions, both graves are similar in that they also resonate well with practices which would eventually become commonplace in the Late Nordic Bronze Age; one might even call them both harbingers of Late Bronze Age cremation deposition patterns.

The remains of the subadult individual from Egshvile, Thisted County (THY 2554, Urn N6) were treated in a notably different fashion than were the remains of the other early cremated remains from Thisted County; the individual from Egshvile was placed in an urn (vs. spread out and presumably wrapped in hides/skin/textiles) within a cist. Because of the placement in an urn, this type of cremated burial has a greater similarity to Late Nordic Bronze Age funerary practices than to other contemporary funerary arrangements. Another cremation grave from the same mound at Egshvile (chronologically speaking dated from an only slightly later part of the Early Nordic Bronze Age) also contained a cremation which seems to be that of a female (Egshvile THY 2554, Urn N5) according to the associated grave goods [42]. A radiocarbon date puts this individual very near to the subadults from Urn N6 (AAR 8827, 3049 +/- 27 BP at 1-sigma confidence level [42]). Unfortunately, no material suitable for Sr analysis was available from Egshvile Urn N5. However, recent metallographic analyses of the copper from a tanged knife from that grave suggest that the copper originated in the region around south Tyrol [81].

Let us juxtapose this data with our other non-local Early Nordic Bronze Age grave from our dataset: Hvidegaard (NM B 9220). Like Egshvile Urn N5, Hvidegaard also contained potential references to distant areas. Within the pouch which accompanied the Hvidegaard grave were found the remains of a non-venomous snake particularly associated in the Mediterranean with Asclepius, the Greek good of healing [82]. Likewise, one must consider Lomborg’s argument regarding the contents of the bag; namely that the squirrel jaw and small stones found within may have been from the stomach contents of a bird of prey, and that this may have been indicative of Etruscan practices of divination through the examination of entrails [43, 83].

But possible links to distant areas within the associated grave goods are not the only thing uniting these two Early Nordic Bronze Age non-local cremations. Our results indicate that the cremated remains from Hvidegaard contained not only the young male (i.e. the individual from whom we have non-local ^87^Sr/^86^Sr), but also a young subadult. Unfortunately, there was no skeletal material from the subadult suitable for strontium analysis. Nonetheless, the presence of this subadult is important in several respects. The overt presentation of the grave from Hvidegaard (NM B 9220) as a single individual (i.e. the cremated human material was placed in a single pile wrapped with wool placed on a cowhide and annotated with objects at appropriate intervals as if it represented only one deceased person) merits particular note. It represents a sharp contrast with the grave from Villerup (THY 1696, N10) which includes the remains of what seems to be a single individual (a possible female) which were divided into two separate and distinct piles. Secondly, even though the Hvidegaard material was not placed within an urn, it nonetheless resembles other tendencies of the Late Bronze Age, namely the likelihood that, should children be cremated, that they were placed together with adults in the same contexts, as has been observed elsewhere in Southern Scandinavia [84, 85].

This combination of adult and subadult within a single context such as is present in the Hvidegaard grave from Zealand allows for comparison with the published results of previous work. The Egtved grave from the Early Nordic Bronze Age (Period II) is a well-known case presenting a combination of an inhumation and cremation: that of the inhumed female (the ‘Egtved Girl’) and an associated subadult cremation from Vejle County in central Jutland (Denmark) [86, 87]. Recent isotope analyses of the human remains of the Egtved female’s burial show that both she and the five-to-six year old subadult who accompanied her had strontium isotope values very similar to each other, suggesting that both individuals originated from the same geographical area, albeit one outside of present-day Denmark [73]. Further contrast to the Egtved grave from within this dataset comes from the combined inhumation and cremation at Erslev (NM 175/29, Grave C). While the Erslev grave contained both an inhumation and a cremation (as well as an additional unburnt cranium), we interpret the results of the strontium isotope analyses of the inhumed individual to be local.

All in all, these new data paint a very complex picture. On the one hand, the majority of Early Bronze Age cremation burials examined here exhibit ^87^Sr/^86^Sr ratios which fit within the established baseline for present-day Denmark (and, potentially, the North Frisian Islands and/or Bornholm). On the other hand, aside from potential links with distant areas and some early resemblances between these graves and later Late Nordic Bronze Age practices, there are few other common denominators between the contexts of the two non-local Early Nordic Bronze Age cremations at Egshvile and Hvidegaard. As these links are tenuous at best, we suggest that the key to beginning to better understand the uptake of cremation may be not in looking at similarities between the graves, such as e.g. the origins of the individuals on whom cremation was first practiced, but rather at the tremendous variations in their depositions instead.

Tim Flohr Sørensen’s examination of the differences between Early Nordic Bronze Age cremation graves in Denmark [9] underscores these important differences. There was no single way in which cremations were completed and the resultant remains deposited. This variation may have contributed to Oestigaard’s observation that “cremation is not one, but many funeral practices” [88].While some of the cremations could be classified as *busta* (i.e. graves in which the deceased was both cremated and buried) [89], others were not. Some cremations included single individuals in single piles of remains, others included single individuals in separated piles within a single context and still others included several individuals or even both humans and animals within single contexts. There may also have been various versions of what constituted part of the body in the arrangement of these Early Nordic Bronze Age funeral pyres *sensu* Sofaer [90]; there seems to have been no strict pattern in relation to which grave goods were included on the funeral pyre with the corpse, and which were included (unburnt) in the eventual deposition (63). Some early cremations were wrapped (e.g. in textiles or hides), while others were placed in urns in a manner resembling more standard Late Bronze Age depositions [9]. Finally, the grave goods associated with these Early Nordic Bronze Age cremations were sometimes laid out upon the cremated remains as if they represented an inhumed body (i.e. according to a body schema), and sometimes were placed without any discernable order [9, 91].

The unstandardized and highly varying treatment of many of these early cremations can be contrasted with recent work detailing the highly standardized placement of objects in contemporary inhumation graves from Vejle County, Denmark [27]. All in all, this suggests that, in some instances, the idea of cremation as a burial practice may have arrived in Denmark without an established *habitus* [92]—in this case, a set way in which cremation was to be enacted. Therefore, the transfer of practices associated with inhumation to cremation as a new funerary practice may represent a kind of skeuomorph *sensu* Knappett [93]. In this way, the deposition of early cremations preserved some of the forms of a previous funeral tradition (i.e. treatment of the cremations as a physical non-cremated body) until their function eventually fell away with the transition to urn cremation as the dominant form of funeral deposition in the Late Nordic Bronze Age.

In order to accurately assess the changes and variations examined within the present research, it is important to have a grasp of the space of time over which they occurred. Models for the divisions of the periods for Danish prehistory were established already in the late 19^th^ century, based largely on rigorous typological work [94]. Although those divisions have been refined over the years (e.g. [95, 96], the boundaries have remained largely the same. For the periods discussed within this paper, the earlier cremations (appearing in Periods II-III) occur over a period spanning 400 years. If we follow an estimate for generation length of approximately 25 years [97, 98], these changes seem to have taken place over an estimated 16 generations. This accounts for a comparatively low tempo of cultural change, suggesting that cultural information and practices from previous periods were preserved and passed on to a large degree [78, 99–101]. Such a long duration supports a pattern of cultural continuity and the integration of new information/methods as opposed to cultural adaptation as a result of demographic change.

### Late Nordic Bronze Age data

This slow and varied negotiation which seems to have taken place with regards to the first cremations in the Early Bronze Age serves as an excellent foil to the more standardized urn cremations which characterize the Later Bronze Age [78], and which we see within our last body of study material from Vesthimmerland. As mentioned above, the results of our provenance investigations on Late Bronze Age material from Stenildgård, Vesthimmerland suggest the presence of both locals and non-locals. However, a closer examination of the data brings further complexities to light.

As a point of reference, we must consider an environmental sample from the Stenildgård area itself (KF1306 plants/roots), which yielded ^87^Sr/^86^Sr = 0.709728 [15]; other baseline data from recent work on bog bodies from the greater Vesthimmerland region provided further environmental samples, whose ^87^Sr/^86^Sr ranged from 0.70809–0.70944 [102]. Accordingly, at ^87^Sr/^86^Sr = 0.710608 and ^87^Sr/^86^Sr = 0.711115 the samples from VMÅ 2883 Urn A349 and VMÅ 2560 Urn A23 may still have been local to present- day Denmark, but may not have been living in the direct vicinity of the Stenildgård area up until the age of two years (which is the period during which the inner periosteal layer of the *pars petrosa* is still being formed). That being said, the distance that the persons from VMÅ2883 Urn A349 and VMÅ 2560 Urn A23 may have travelled may not have been very great—similar values offering other potential geologies on which these individuals may have spent their childhood can be found within some few tens of kilometers’ distance.

If we compare this data with extant provenance work on Late Neolithic Stenildgård [15], it seems as if the inhabitants of this area continued the practice of cremation exclusively on non-locals over a significant amount of time. When we examine that data more closely, however, some interesting differences emerge. See Table 7. We can see that the persons on whom cremation was practiced present dissimilar ranges of non-local ^87^Sr/^86^Sr. The four Neolithic samples’ ^87^Sr/^86^Sr ranged from 0.711437–0.711825 [15], which suggest origins which are both outside the local baselines established for the Stenildgård area [15, 102] as well as for present day Denmark [15, 20, 64–71, 102]. In addition, the ^87^Sr/^86^Sr from the Late Neolithic non-local dataset is very closely grouped (samples KM113, KM214 and KM216 are all ^87^Sr/^86^Sr = 0.7114 and samples KM113 and KM214 are both ^87^Sr/^86^Sr = 0.71146). By comparison, the range exhibited by the Late Bronze Age samples from the nearby mounds examined in the present research is somewhat wider, which follows along with a recent large study of migration patterns within Denmark from the Late Neolithic through to the Early Iron Age [17]. While our Late Bronze Age provenance data from Stenildgård also suggest that the cremation samples represent non-local individuals, it also suggests that the origins of those non-locals may have been more varied (i.e. both from outside present-day Denmark as well as from within present-day Denmark, but outside the local region) than the recently published Late Neolithic data sampled nearby.

**Table 7:** Table showing comparative grouping of present data from Stenildgård, Vesthimmerland together with recent analysis of a nearby Late Neolithic grave from the same region [15].

Group	^87^Sr/^86^Sr Range	Sample No.s	Interpretation
A	0.7080–0.7097	(None)	Local to Vesthimmerland
B	0.7081–0.7111	KF 1839 (LBA)	Local to Denmark
		KF 1840 (LBA	
C	0.7111–0.7122	KM113 (L Neo)	Non-local to Denmark
		KM214 (L Neo)	
		KM215 (L Neo)	
		KM216 (L Neo)	
		KF1837 (LBA)	
		KF1838 (LBA)	
		KF1841 (LBA)	
		KF1842 (LBA)	
		KF1844 (LBA)	
D	0.7120–0.7140	KF 1843 (LBA, boar)	Non-local to Denmark

### Data from the faunal material

The one outlier in the Late Nordic Bronze Age material seems to be the boar’s tusk from Stenildgård (VMÅ 2560 x133 Urn A22), due to its very high ^87^Sr/^86^Sr value (^87^Sr/^86^Sr = 0.714090). This contrasts sharply with the two other values measured from the human material from that selfsame urn (a *pars petrosa*, whose ^87^Sr/^86^Sr = 0,711516 and a second molar, whose ^87^Sr/^86^Sr = 0,711768, which may both come from the same individual). As boar’s tusks have symbolic connections with high status males and warriorhood both in Scandinavia as well as the greater European Bronze Age [103, 104], we suggest that the tusk may represent e.g. a trade item or an object picked up while on a hunt/travels abroad.

This datapoint may be contrasted with the other faunal sample analyzed within this study, namely the *ovid/capra* tooth from the Early Nordic Bronze Age grave from Nørhågaard (THY 1550, Grave N3). Our study revealed that the sheep/goat yielded ^87^Sr/^86^Sr = 0.71041. This ratio is very close to that measured from the possible female cremation with whose bones it was mixed (which yielded ^87^Sr/^86^Sr = 0.71040. Within their much larger study of 3^rd^ and 2^nd^ millennia BC material, Frei et al. [17] had previously conducted strontium isotope analyses of the inhumation at whose feet the cremated remains of this possible female were placed. In their article, the authors conclude that the Nørhågård inhumation represents that of a probable male individual with ^87^Sr/^86^Sr = 0.71046 [17]. The results of these two individuals and the cremated animal from Nørhågard are very similar, which may suggest that all three where from the very same area. Despite the differences in the nature of the depositions (i.e. inhumation vs. cremation; female vs. male, human vs. animal), the strontium results intimate a very tight connection of both humans and animal in terms of geographic provenance.

### Comparison with other published cremation data

To return exclusively to human remains, however, the many cases of potential Early Nordic Bronze Age locals observable both from Thisted County as well as from the site of Maglehøj in Zealand contrast sharply with the Late Nordic Bronze Age material from the Stenildgård area in Vesthimmerland, which exhibits an apparent continual deposition of foreigners, both within its earlier (Late Neolithic and Early Nordic Bronze Age) funerary depositions [15] as well as within the Late Nordic Bronze Age material examined within the present study. Review of published data from other Late Nordic Bronze Age to Early Iron Age cremation contexts, however, intimates that the data from Stenildgård in Vesthimmerland may not be indicative of a general trend. Although their study was generally more focused on testing the validity of strontium isotope analyses on cremated remains, Harvig et al. nonetheless produced provenance data on a further twelve individuals from chronologically comparable contexts [32]. These data included one adult male individual from a Late Nordic Bronze Age urn cremation from Rishøj near Viborg on Jutland and eleven Late Nordic Bronze Age cremation urns /Early Iron Age cremation pits from four sites from Fraugde Parish on Fyn. While the Late Nordic Bronze Age individual in the cremation urn from Rishøj exhibited ^87^Sr/^86^Sr outside the range of present-day Denmark (^87^Sr/^86^Sr = 0.71159), the provenance data from Fraugde Parish suggests the presence of at least two persons interpreted as non-locals [32].

### Discussion Nordic Bronze Age as a whole

All in all, these data suggest that both locals and non-locals seem to have been cremated in the Nordic Bronze Age. Upon further study, we suggest that the spatial variations within migration patterns observable within the cremation data may be indicative of differences in the mode and tempo of cultural change in the regions concerned. The presence of such eddies within the increasing upsurge of social changes which took place across Denmark throughout the Nordic Bronze Age have already been postulated within other research contexts [36, 38, 105–107]. Recent discussion of Thisted County in particular stress that this region was likely to have had a period of economic boom and population influx from 1500–1100 BC [81]. However, as we have seen from the strontium isotope data here, our samples from the cremation graves from this period suggest a highly local population.

There is one area in which all graves from all sites, study areas and time periods examined by the current research are the same: an apparent association with high social status. The stratification of Nordic Bronze Age society is thought to have been expressed through high-status elites buried in mounds surrounded by varying prestige goods obtained through long-distance alliance systems; this understanding has long been a mainstay of scholarly conceptualizations of this period [22, 38, 39, 105, 108–110]. The graves of such high-status individuals can be contrasted with the graves of so-called ‘commoners’, which are thought to have been organized in nearby flat cemeteries [16]. However, the day-to-day reality of elite society in the Nordic Bronze Age was likely on a vastly different scale than that with which we are familiar in more modern times. It has been posited to have taken the form of local chiefdoms [38, 105] maintained by local chiefs/big men with social, political, trade and kinship connections with other chiefdoms [39, 40, 107, 111, 112]. The presence of the estimated 40–50,000 grave mounds [113, 114] associated with these elite chiefs across Denmark (and which, in many cases, were used for multiple burials) gives excellent perspective in terms of scale. For this reason, in addressing status in relation to cremation (and its variations), it is very much likely a question of degree; small differences and divergences may have been highly important, and may even have spurred on the adoption of cremation.

In order to potentially evaluate status in relation to these cremations, we need to delve a little more deeply into how cremations were executed in prehistory. The cremation of a human body is a feat which is not easily executed [115, 116]. In the Roman period, the professional attendants of cremation were called *ustores* [117]. It follows, therefore, that, especially in the first few instances of its execution, a funeral by fire may have been an arresting process to witness. Such a practice would lend itself well to a reaffirmation of elite status. As a performance in support of elite status, the early uptake of cremation would dovetail well with Miller’s work on the manner in which elites continually seek to re-define their class through changes in style [118]—in this case, through the adoption of a new funerary practice.

Addressing style in relation to the integration of cremation brings to the fore a very important point. In examining the uptake of cremation through provenance analyses, the underlying assumption is that we will shed light on the likelihood that the idea of cremation may have been first introduced by non-locals (or not). However, as intones the oft-cited archaeological adage, “the dead do not bury themselves” [119]. Discussions about contemporary funeral practices throughout Europe have mooted the idea that burial modus and grave goods were a better reflection of the burying party rather than they were of the deceased [120–124]. Ethnographers and anthropologists propose that funerals are objects of “collective representation” [125]; furthermore, they were also occasions upon which the collective social order was renegotiated [126–128]. Therefore, we must consider the possibility that the social importance of the early cremation graves may have been less a reflection of the deceased (whatever their local/ non-local status) and more a tacit reminder of the changing social environment in which the deceased was put to rest. As was pointed out by Oestigaard and Goldhahn [30], the mere shift to cremation (vs. inhumation) may have also allowed for a bit more room to maneuver in terms of organizing the sort of grandiose funerals which might have accompanied the death (and the subsequent social re-ordering) associated with a sudden absence in the elite sphere. This same phenomenon was also noted by Mauss, albeit in relation to funerals in general rather than cremations specifically [129]. By switching to cremation, the burying community may have extended the period during which they could arrange a larger social gathering (possibly also from a greater array of enclaves both near and far [30]). After all, the princely burial at Hochdorf from Late Hallstatt Baden-Württemberg in Germany (540–520 BC) took an estimated five years for construction [130, 131].

If we consider that the utilization of a visually-arresting new funerary practice may have been an attempt to improve the status and/or socio-political connections of the surviving members of the funeral party through the rich burial of another (deceased) member of their group, it would behoove us to remark upon the cremation burials of the infant at Villerup (THY1696, Grave N10), the two young subadults at Ginnerup (THY5055, Grave N16), the subadult at Egshvile (THY 2554, Urn N6) and the subadult buried together with the young male at Hvidegaard (NM B 9220). The tender ages of these cremated individuals may be evidence for other social machinations surrounding the funeral (e.g. aggrandizement of the standing of the burying party and/or peripheral re-negotiations of the alliances of those attending). This is particularly remarkable when contrasted with the fact that both in Egshvile and Villerup, the mounds were later extended to contain the graves of adult individuals belonging to the warrior class, presumably from the same social grouping. At each site, one of the secondary graves contained objects evidencing contacts with the outside world: in Egshvile, the richly equipped female had glass beads, and in Villerup, a man was buried with a Norddorf-type pin as well as a sword [14].

In conclusion, in addition to underscoring the need for more data, the obvious variations in the Early and Late Nordic Bronze Age data refute the possibility of presenting a single blanket scenario describing the uptake of cremation across Denmark. As a result, we present the following scenarios as potential methods for the arrival and uptake of cremation on Danish shores in the Early Nordic Bronze Age:

1. A scenario in which Early Nordic Bronze Age persons began to cremate their dead independently of established cremation traditions within contemporary cultures in other parts of Europe (so-called ‘homoplasy’ [132, 133]).
2. A scenario in which both non-locals and local people (or persons from geologically-similar areas with similar cremation establishment timelines) may have been the first to practice cremation. Although the ^87^Sr/^86^Sr range of the individuals analyzed from Villerup, Erslev, Nørhågård and Ginnerup (^87^Sr/^86^Sr = 0.70989 to 0.71099) from Thisted County and Maglehøj (NM B 4092–95) from Zealand (^87^Sr/^86^Sr = 0.71095) do fall within the range of what may be considered local for present-day Denmark, they also fall within the baseline range of other areas of the Bronze Age world in which cremation experienced an early adoption, such as the Frisian Islands and Bornholm (see discussion cremation [14, 77, 78]). Hence, it is possible that some (or all) of the ‘local’ individuals were coming from e.g. the Frisian Islands, Bornholm or other parts of Denmark outside the areas which we sampled in this research.
3. A scenario in which the concept of cremation may have been brought back to Denmark by returned (possibly local) travelers who had witnessed cremation abroad.
4. A scenario in which cremation was integrated as a secondary effect of the inter- and intra-regional social re-negotiations which have been postulated as a result of elite death.

Seen together, we suggest that the integration and establishment of cremation as a new burial practice within the Early and Late Nordic Bronze Age material described above suggests that there may not have been a one-to-one relationship between immigration to Denmark and the uptake or continuation of cremation. Instead, the results of these present investigations can be coalesced into three important observations. Firstly, the adoption of cremation as a practice and its eventual cultural entrenchment may have been highly dependent upon the socio-political and economic pressures of the various regions. Secondly, the very nature of cremation itself allowed for a more drawn-out funerary process, thereby potentially enabling the convocation of kinship and alliance partners both near and far and the socio-political jockeying which would ensue. Finally, the arrival of cremation without an established *habitus* suggests that the integration of that new funeral practice was itself constantly under negotiation in the early years of its introduction. This last point seems to be supported by a large amount of archaeological evidence. The presence of both local and non-local individuals within both the Early and the Late Nordic Bronze Age cremations nonetheless intimates that both locals and non-locals were cremated according to would eventually become an integrated tradition within Denmark by the later part of the period.

## Conclusion

This study presents the results of strontium isotope analyses from Early Nordic Bronze Age sites from Thisted County (Jutland) and the island of Zealand and Late Nordic Bronze Age sites from Thisted County and Vesthimmerland (both from the Jutlandic peninsula). The new data include samples from the sites of Villerup, Egshvile, Erslev, Nørhågård and Ginnerup (all from Thisted County), Hvidegaard and Maglehøj from Zealand and from several different sites in the Stenildgård area in Vesthimmerland. These 19 new data points comprise both Early and Late Bronze Age contexts, cremated human material as well as one inhumation (where that inhumation was included in direct relation to cremated human material) and two instances of cremated faunal material deposited alongside the cremated human remains. With the exception of the individuals tested from Egshvile (Thisted County) and Hvidegaard (Zealand), all other Early Bronze Age individuals exhibited strontium ratios within the baseline for present-day Denmark. By contrast, with the exception of Ginnerup (THY 5055, Grave N30) from Thisted, all of the Late Bronze Age samples (including all of the sampled material from the Stenildgård region of Vesthimmerland) exhibited strontium ratios which were either outside the baseline for present-day Denmark or outside the baseline for the local region of Vesthimmerland.

Analysis of this data in relation to considerations of current archaeological research, other published provenance data and particularly the specific contexts of the graves examined has lead us to propose a series of four potential scenarios which may have brought about the introduction of cremation as a burial practice to Denmark within the Nordic Bronze Age. The data also suggests that the mechanisms bringing about the integration of cremation as a new burial practice may have differed from one region to another. Our analyses intimate that the long durations of funeral depositions made possible by cremation may have lent themselves particularly well to the kind of social renegotiations which social theorists suggest follow upon the heels of elite death. Furthermore, the lack of an established *habitus* associated with the early instances of Nordic Bronze Age cremations recorded in the archaeological record point towards a continual negotiation with ideas rather than the introduction of extant ideas through wholescale population turnover. This can be contrasted with an established way of doing cremation in e.g. the Late Nordic Bronze Age, which was more standardized.

While we await the results of future analyses in order to further solidify the observations above and to determine which of the proposed scenarios is most likely, it is important to note that there are several new avenues which that research may take. For example, one might consider a more archaeological approach rather than an archaeometric one. Further investigations might take the form of a critical cross-comparison between the funerary traditions of our study areas with those of other regions with whom the inhabitants may have had trading, socio-political and/or kinship ties (e.g. Thisted County and Norway [107]). It is certain that there are other examples of transitional burial skeuomorph cremations, including Gundsømagle (Ke 453) [134], Jyllinge (Ke 469) [134] and Tjæreborg (Ke 4120) [135].

Nevertheless, the variations in the data presented thus far refute the possibility of presenting a single blanket migration scenario describing the uptake and establishment of cremation across Nordic Bronze Age Denmark. While the observations of the New Mobilities Paradigm are certainly correct insofar as ideas cannot be transferred across pre-literate societies without human carriers [11, 136], our data nonetheless do not suggest a direct, one-to-one relationship between in-migration and the adoption of cremation within the area of present-day Denmark. Further research may help us to unravel the complex mechanisms which governed the uptake of this new idea and its eventual establishment as the norm of the Later Nordic Bronze Age. In this way, these new data add an intriguing new layer to our evolving vision of Nordic Bronze Age society while also leaving us with many new questions and avenues of research through which to examine the social and cultural dynamics within this important and rich enclave of Nordic prehistory.

## Supporting information

S1 Fig (TIF)

S2 Fig (TIF)

S3 Fig (TIF)

S4 Fig (TIF)

S5 Fig (TIF)

S6 Fig (TIF)

S7 Fig (TIF)

S8 Fig (TIF)

S9 Fig (TIF)

S10 Fig (TIF)

S11 Fig (TIF)

S12 Fig (TIF)

S13 Fig (TIF)

S1 Appendix In-depth descriptions of archaeological contexts. (DOCX)

S2 Appendix Osteological analyses and results. (DOCX)
