# Supplementary figures and images for: Into the fire: Investigating the introduction of cremation to Nordic Bronze Age Denmark: A comparative study between different regions applying strontium isotope analyses and archaeological methods
Source: PLoS One. 2021 May 12;16(5):e0249476. doi: 10.1371/journal.pone.0249476 (PMC8115792; doi:10.1371/journal.pone.0249476)

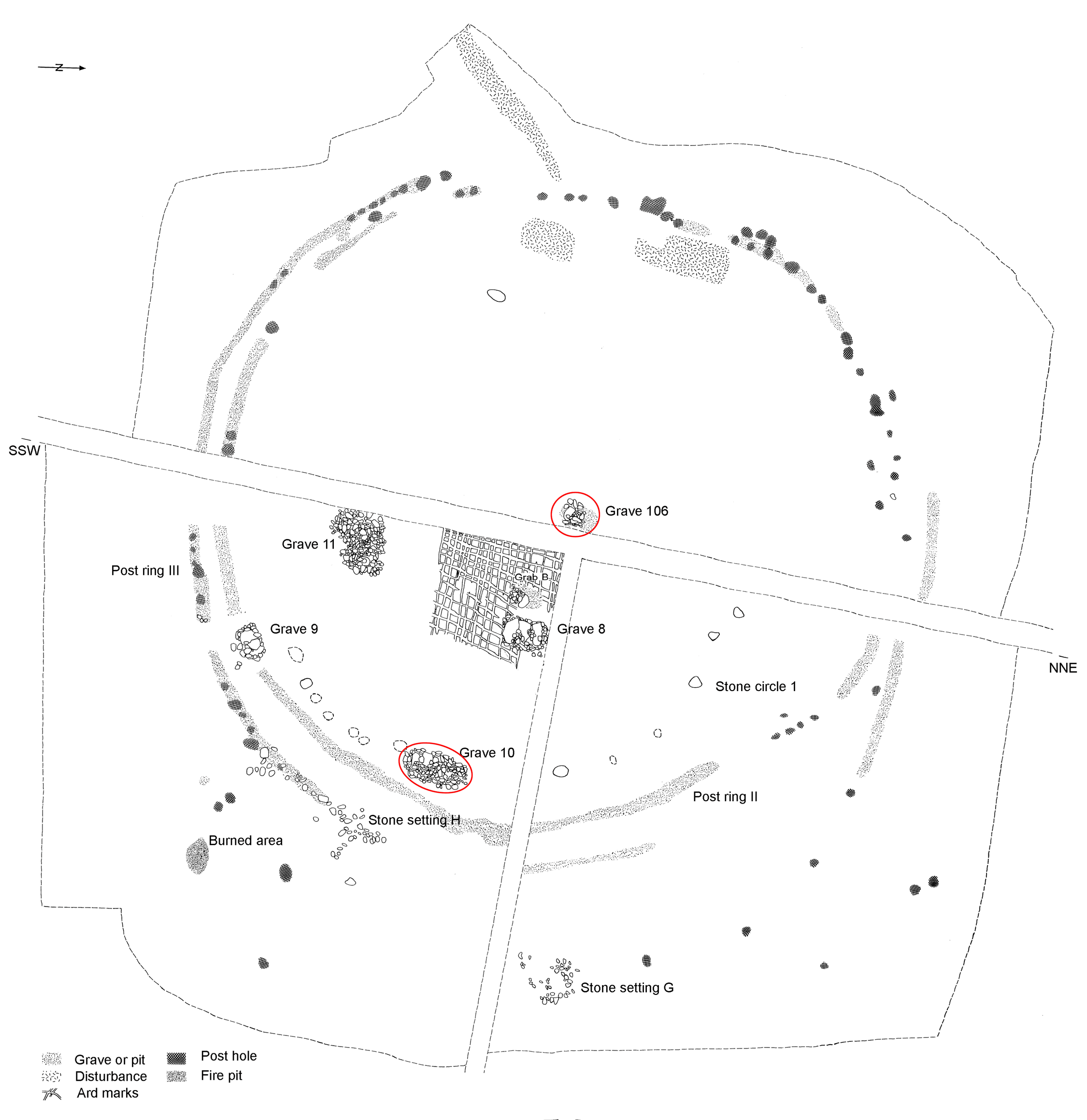

Supplement: S1 Fig — (TIF) [file pone.0249476.s001.tif]

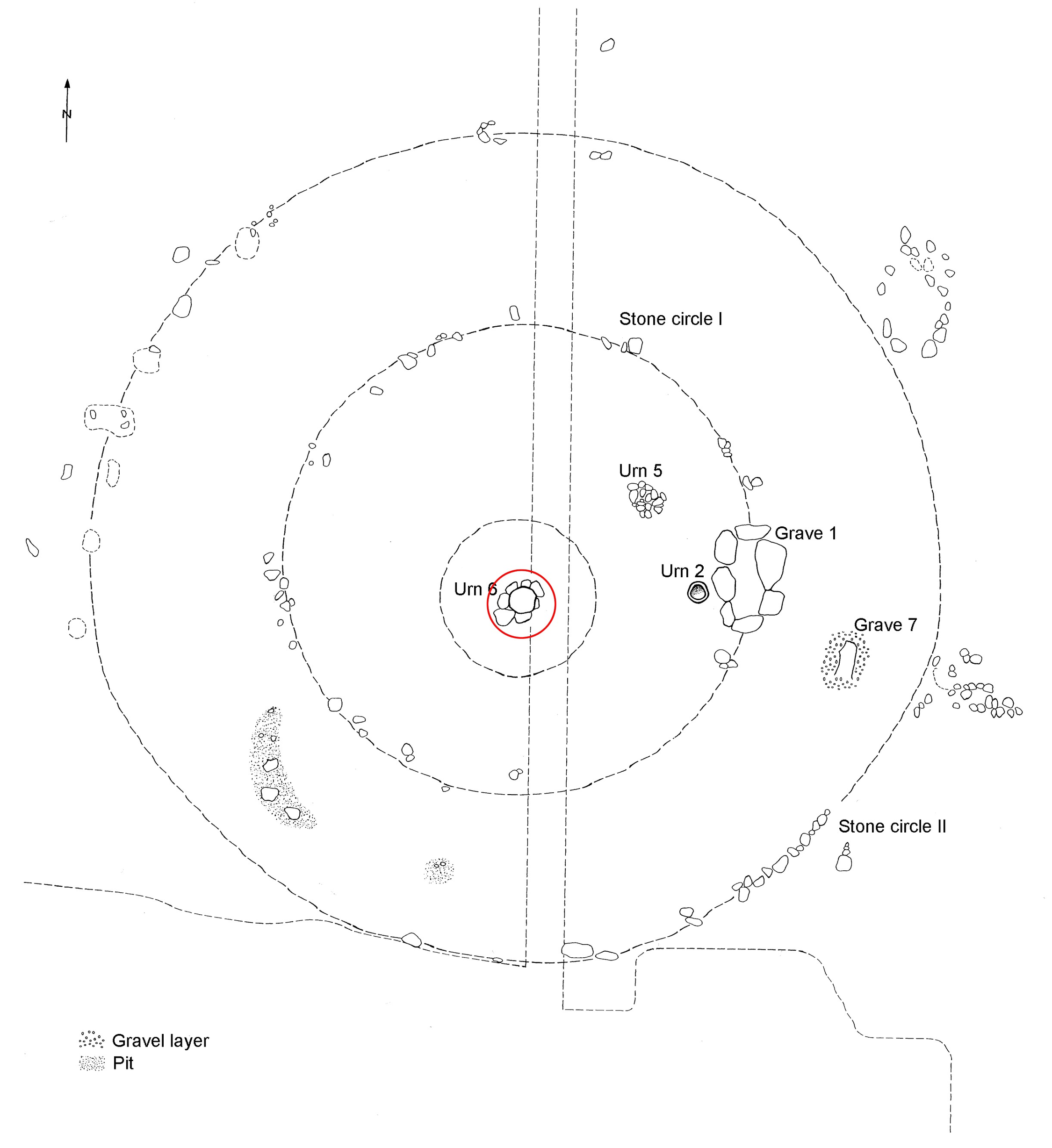

Supplement: S2 Fig — (TIF) [file pone.0249476.s002.tif]

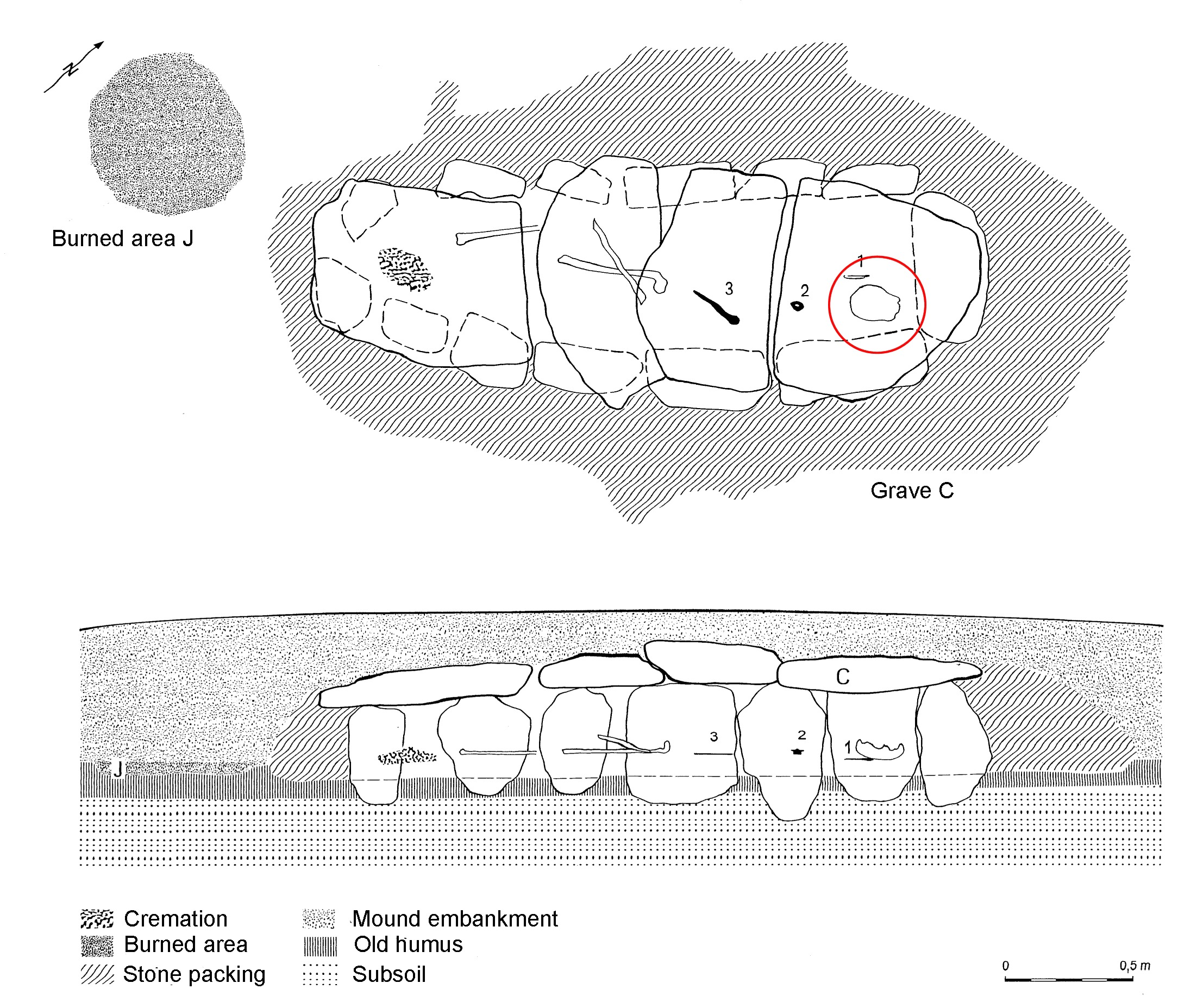

Supplement: S3 Fig — (TIF) [file pone.0249476.s003.tif]

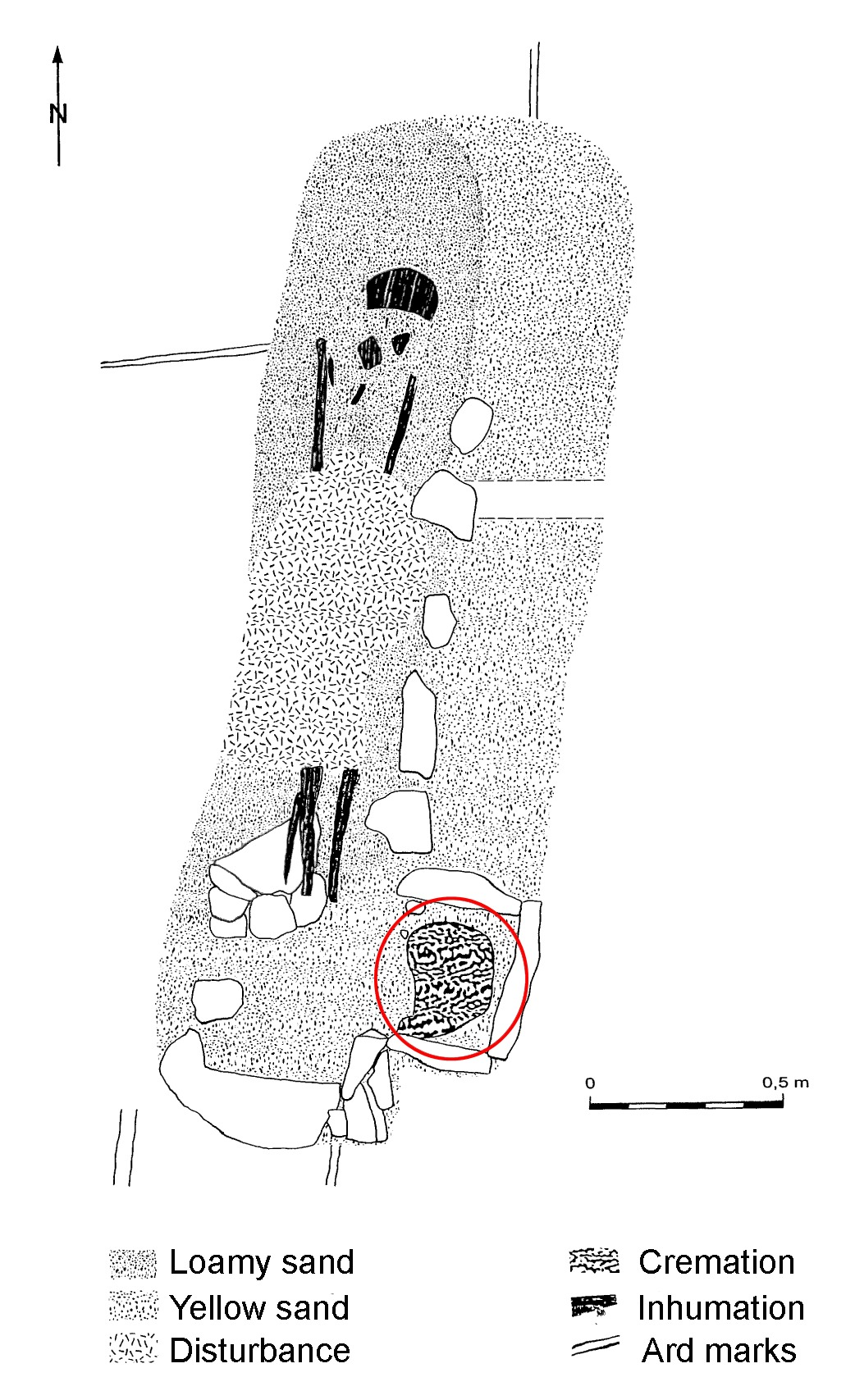

Supplement: S4 Fig — (TIF) [file pone.0249476.s004.tif]

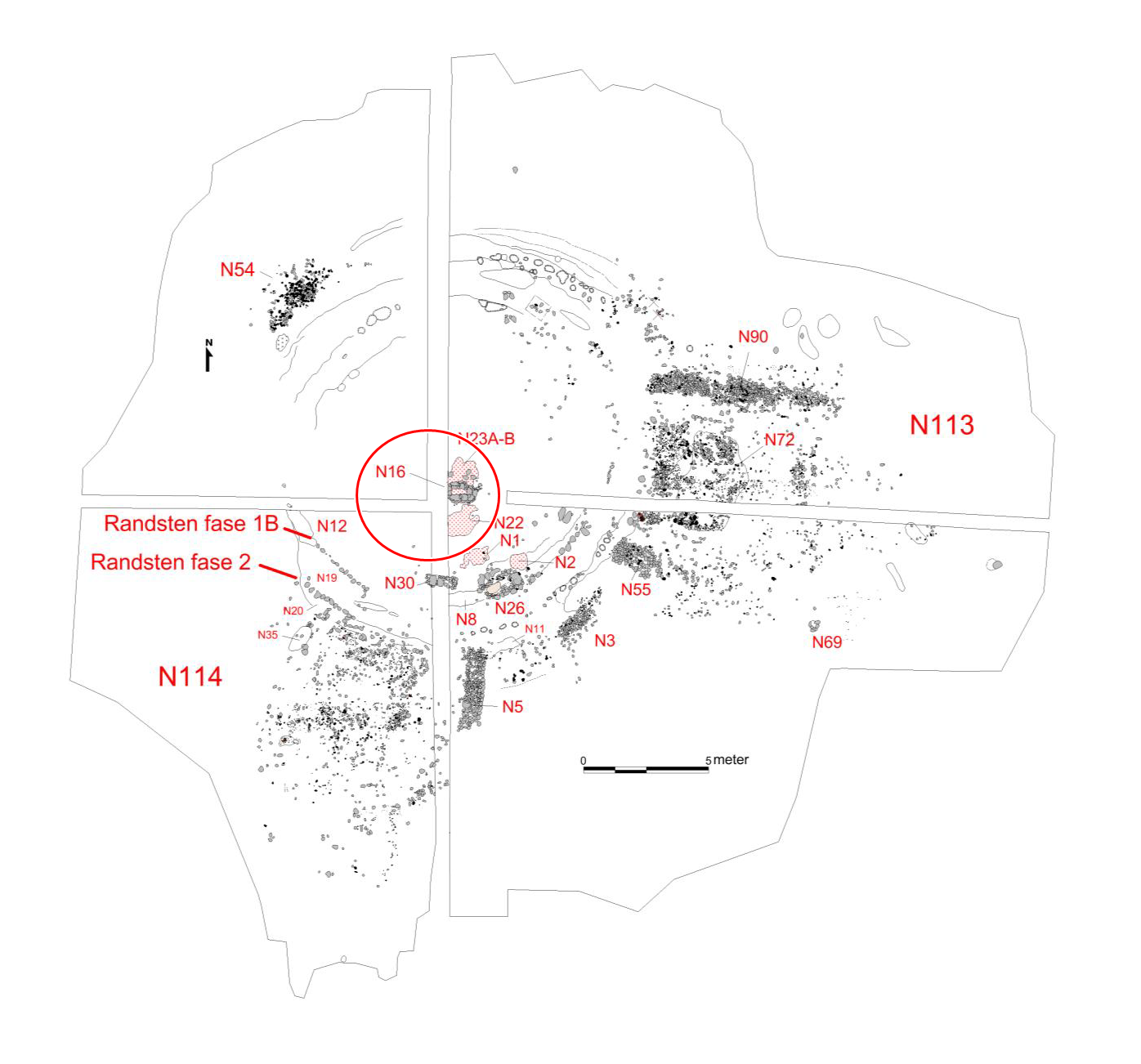

Supplement: S5 Fig — (TIF) [file pone.0249476.s005.tif]

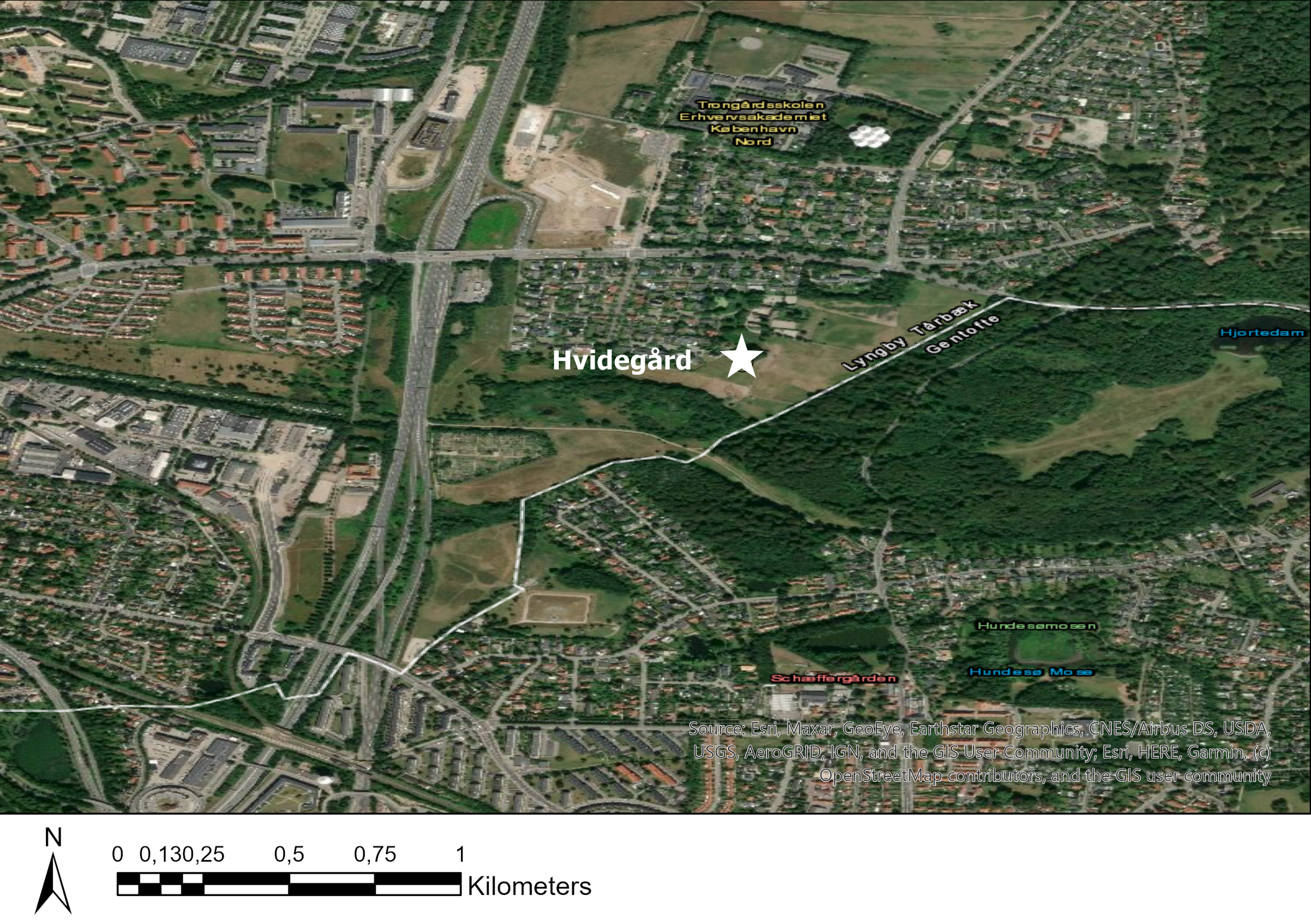

Supplement: S6 Fig — (TIF) [file pone.0249476.s006.tif]

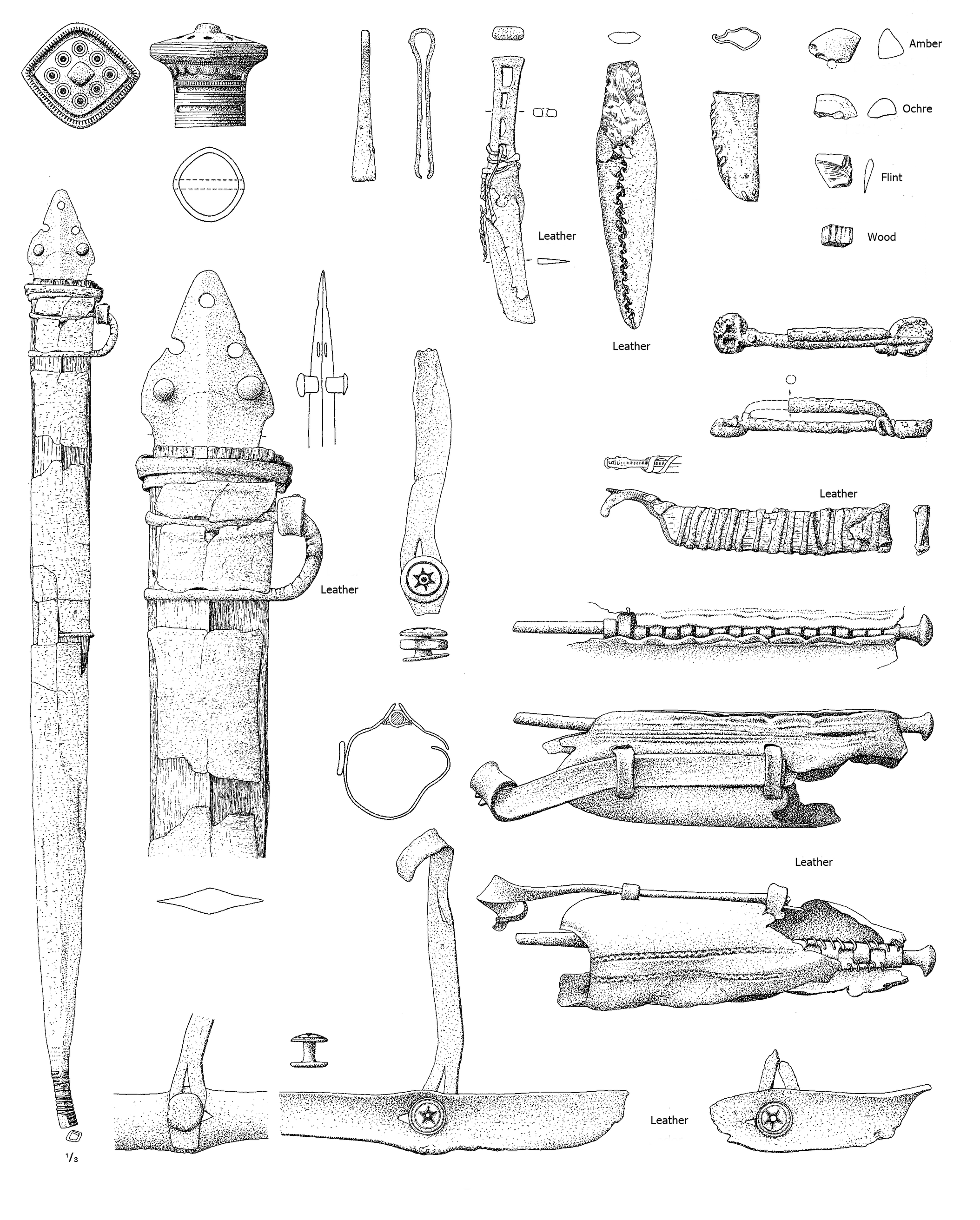

Supplement: S7 Fig — (TIF) [file pone.0249476.s007.tif]

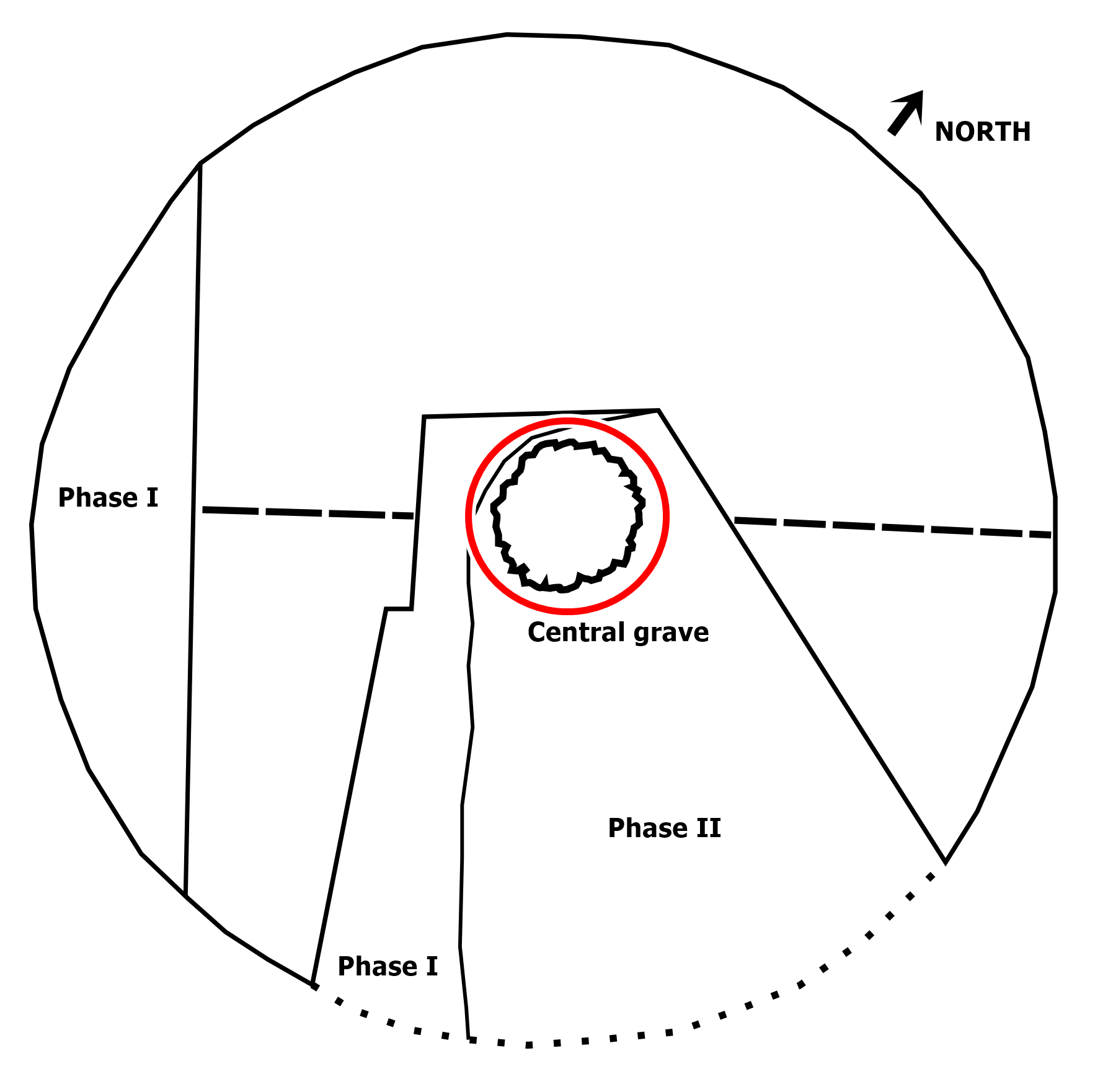

Supplement: S8 Fig — (TIF) [file pone.0249476.s008.tif]

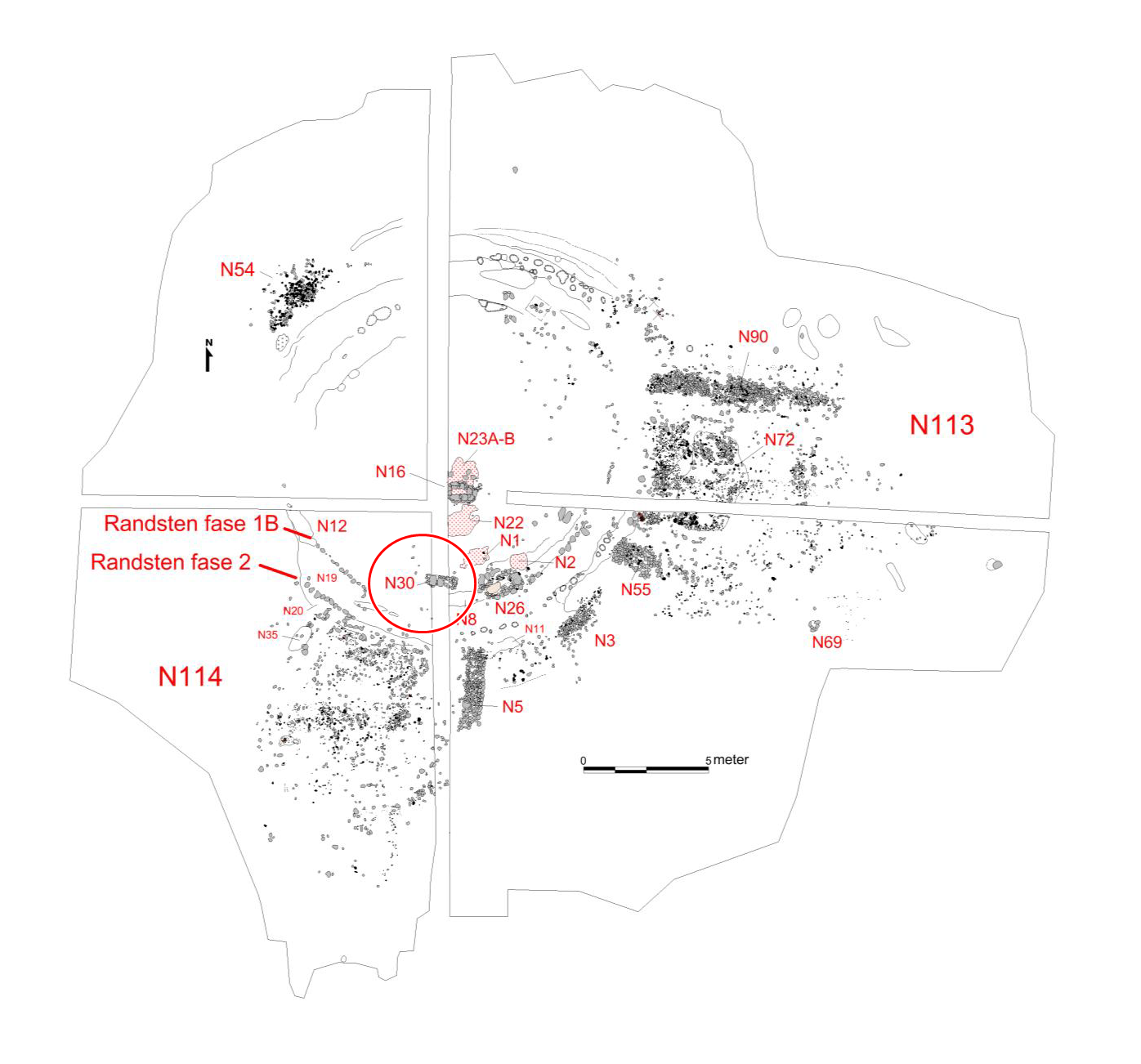

Supplement: S10 Fig — (TIF) [file pone.0249476.s010.tif]

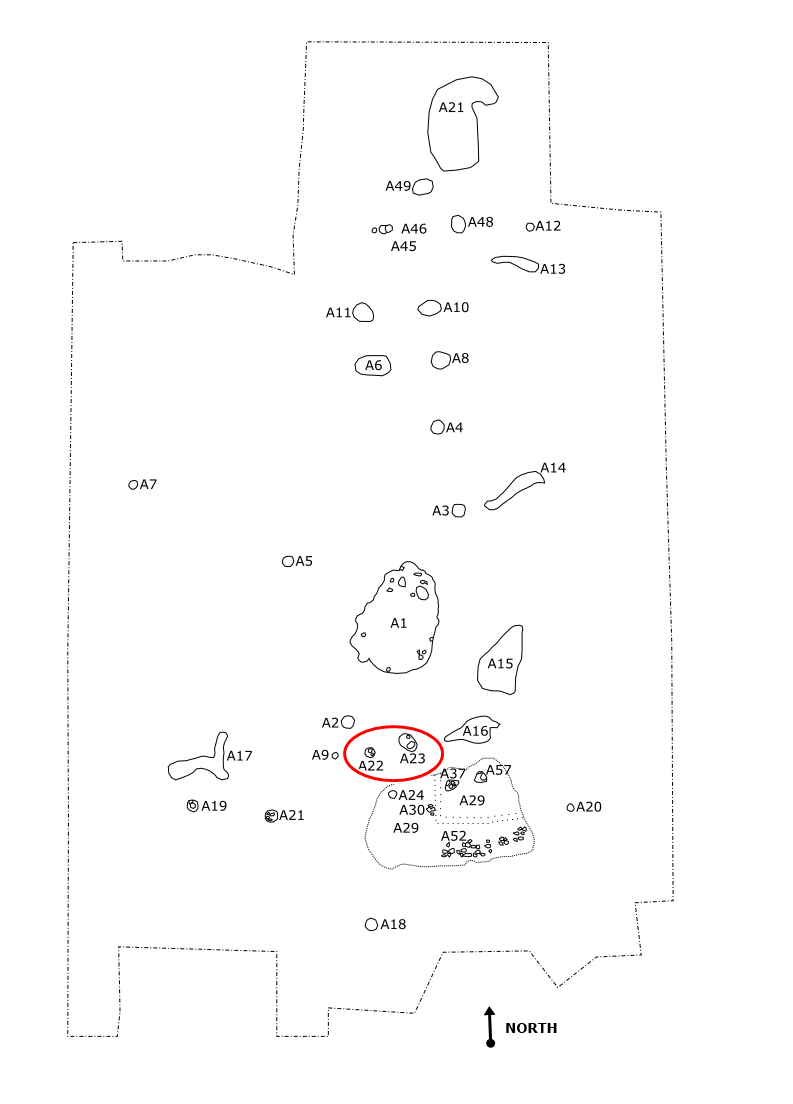

Supplement: S11 Fig — (TIF) [file pone.0249476.s011.tif]

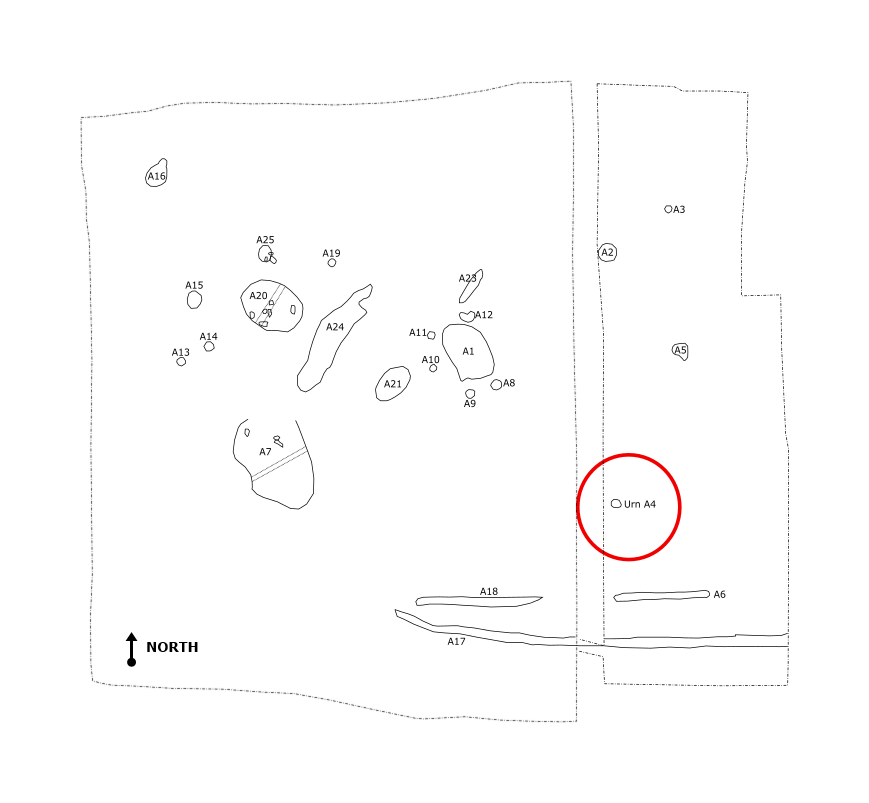

Supplement: S12 Fig — (TIF) [file pone.0249476.s012.tif]

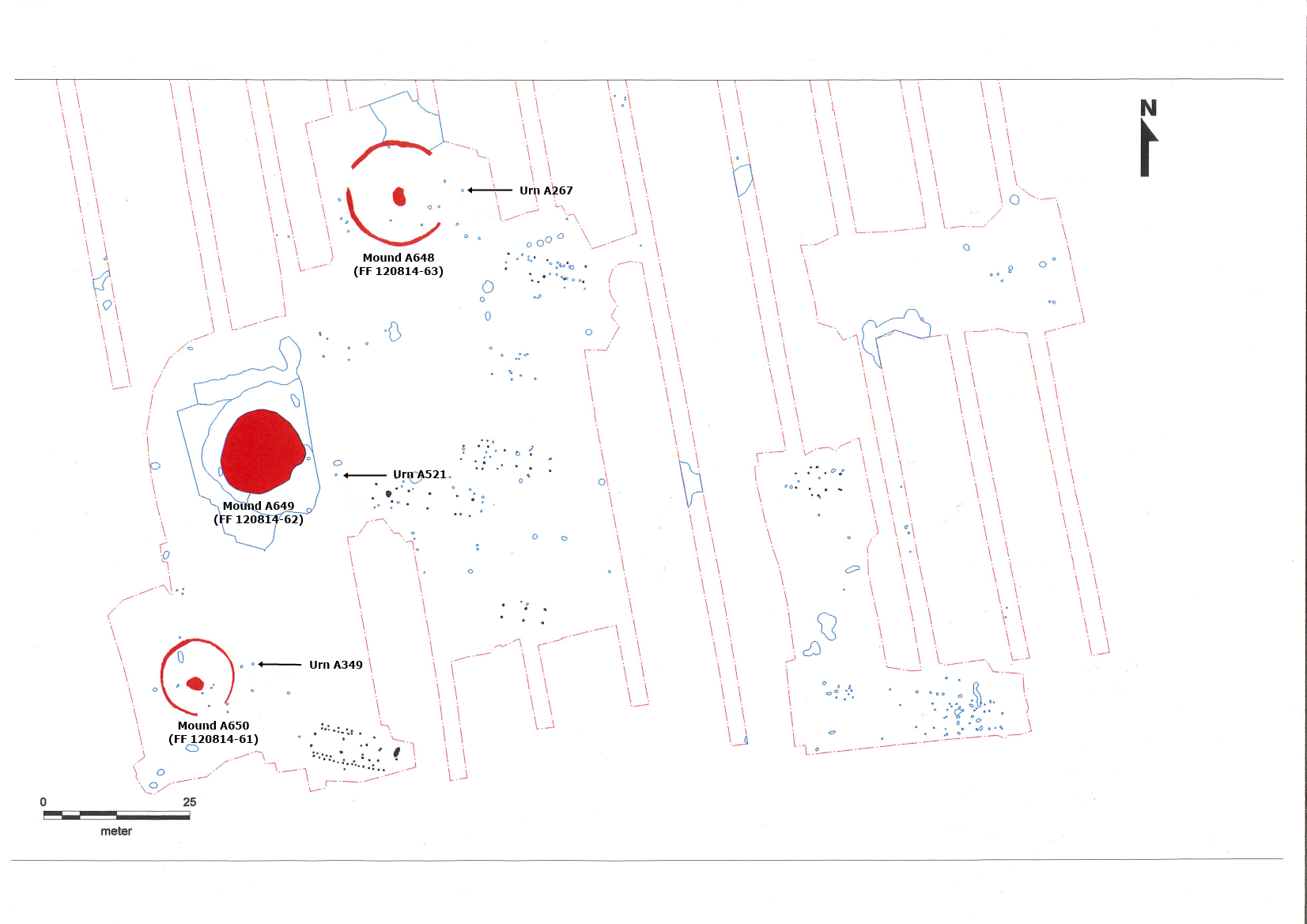

Supplement: S13 Fig — (TIF) [file pone.0249476.s013.tif]
