# Supplementary material for: Into the fire: Investigating the introduction of cremation to Nordic Bronze Age Denmark: A comparative study between different regions applying strontium isotope analyses and archaeological methods
Source: PLoS One. 2021 May 12;16(5):e0249476. doi: 10.1371/journal.pone.0249476 (PMC8115792; doi:10.1371/journal.pone.0249476)
Supplement: S1 Appendix — (DOCX) [file pone.0249476.s014.docx]

# **S1 Appendix. In-depth descriptions of archaeological contexts**

## **Early Nordic Bronze Age Thisted County:**

### **Villerup (Ke 5501, FF 110612-86)**

### The partly overploughed mound at Villerup (THY 1696), was excavated by Museum Thy in 1981-1982 [1]. The mound at Villerup is part of a series of near super-imposed mounds situated on a ridge close to the ancient coastline. Excavation of the mound revealed a total of six stone cists. Prehistoric interventions at the site seem to have even continued into the Early Iron Age. For the present study, we sampled material from two of the Early Bronze Age cists: the central grave (THY 1696 grave N106) from the first mound and another grave from the periphery of one of the larger mounds built on top of it (THY 1696 grave N10).

Alongside the remnants of the pyre-pit it contained within it, the central grave (THY 1696 grave N106) included a thin layer of cremated human remains, a small piece of textile remains, the foot of a sheep/goat into which a hole had been drilled and, importantly, a small bronze tutulus, which could be typologically dated to Period II [2,3]. Physical anthropological analysis of this cremated material showed that it included those of an infant aged from 7-13 months [3]. For the current research, one deciduous molar from this sub-adult individual (THY 1696 grave N106) was sampled.

As mentioned, a further sample was also taken from another cremation grave found at the periphery of the larger mound built on top of the first. This other grave (THY 1696 grave N10) included a stone cist in which two distinct piles of cremated remains were found. In the northernmost of these, excavators found a bone needle and a flint scraper, while in the southernmost they recovered a spiral armring. These objects suggest a broad date to the Early Bronze Age. The preservation of the cremated material from this grave was not ideal; therefore, it was not anthropologically analyzed. However, it has been suggested that the cremation was that of a female individual, due to the grave goods associated with it [2]. For the present research, an adult first premolar was sampled from this individual (THY1696 grave N10).

### **Egshvile (Ke 5115, FF 110210-12)**

In the winter of 1988-1989 a private person noticed a collection of large stones on the surface of an overploughed mound at Egshvile, (THY 2554). This led to the subsequent excavation of the mound by Museum Thy in March and September of 1989 [2,4]. The mound contained three phases, each with an Early Bronze Age cremation burial, and secondary graves from the Late Bronze Age. The subject for the present research is the central grave of the oldest phase of the mound which measured 3 m in diameter and was originally approximately 0,8 m high. The grave contained an urn set in a small stone cist. The urn held the cremated remains of a five-year old child (THY 2554 urn N6, x 18) alongside one complete and one partial amber bead as well as 20 pieces of unworked amber and the leg bones of a calf [4]. Cremated bone from the grave has been ^14^C dated to 3052 +/-46 with one sigma [5]. Hornstrup places the grave in Period II. For the present research, a first molar was sampled from this individual (THY 2554 urn N6, x18).

The mound contained two more cremation graves from the Early Bronze Age, including another urn grave belonging to a 30-50 year-old, richly equipped female from Period II [5,6]. The grave was ^14^C dated to 3049 +/-27 BP at the level of one sigma certainty (AAR-8827) [5]. Unfortunately, there was no available human material suitable for strontium analysis from that grave.

### **Erslev (Ke 5278, FF 110405-26)**

The mound at Erslev (NM 175/29) was excavated by Brøndsted in 1929 [7]. The site included a minimum of eleven individuals, all thought to date from the Early Nordic Bronze Age. However, this paper has chosen to focus on the remains found in a multiple burial cist located at the centre of the mound (NM 175/29, grave C). This central grave contained the partial remains of three individuals: one inhumation, the unburnt cranium of a possible sub-adult and a pile of human cremated remains. Both the cranium and the cremated remains were placed at the feet of the inhumation [7,8]. A nearby fire feature may have been associated with the cremated remains included in the central grave. The present study analyzed one of the second molars from the inhumation (NM 175/29, grave C). The grave goods of this individual consisted of a fibula, a dagger blade and the remains of the pommel of another dagger [2]. These objects can typologically situate the grave within Period II. No anthropological analyses have been completed thus far.

### **Nørhågård (Ke 5176, FF 110304-78)**

As was the case with many of these sites, the overploughed mound at Nørhågård (Hundborg Herred, Nørhå Sogn, THY 1550) seems to have been utilized in both the Early and the Late Nordic Bronze Age. Like the location described above, excavations at the (by then, levelled) mound at Nørhågård conducted by Museum Thy in 1982 revealed another multiple-individual grave containing both inhumed and cremated remains. THY 1550 grave N3 presumably contained an oak coffin covered by flat chalk stones. A supine individual was inhumed in the lower part of an oak coffin. At the foot of the cist, a small niche was offset to the east for the inclusion of cremated human and faunal remains [9,2]. The inhumed individual from THY 1550 grave N3 was analyzed in a previous study (Rise 106) by one of the present authors [10]. The present study sampled the cremated remains of a human *pars petrosa* as well as an ovid/capra tooth found in the cremated material from the offset niche (THY 1550, grave N3, AS 356/2001 and AS 31/94). Anthropological analyses of the human material suggest that the individual from the cremation was an adult and possibly that of a female individual [11]. Typological dating of the niche portion was unfortunately not possible due to lack of artefacts. However, Frei et al.’s analyses suggest that the inhumation with which the cremation was associated was a possible male aged 25-35 years; two ^14^C dates suggest Period III (OxA-28991, 2949 +/- 28 and OxA-28992, 2943 +/-28) [10]. Unfortunately, although cremation graves N4 and N5 from Nørhågård also date from the Early Bronze Age, we were unable to find material suitable for strontium analysis from them.

### **Ginnerup (FF 110605-58)**

In 2008, Museum Thy excavated an overploughed mound at Ginnerup (Refs Herred, Heltborg Sogn, THY 5055, FF 110605-58) [12]. From this site, two individuals were sampled from two different graves. The over-ploughed mound included a central stone cist containing the cremated remains of two children with the remnants of a double pyre-pit. During field excavation the content inside the central stone cist was taken up as a block and excavated at the conservation department at the National Museum of Denmark in 2010-2011 [13]. Excavation showed that the burnt bones were probably wrapped in a very badly preserved wool textile. There were no other grave goods. The children were aged by Lynnerup and Harvig at four and eight years, respectively [12]. Two deciduous teeth from the four-year-old child (a mandibular deciduous first molar and a maxillar first incisor) were sampled from this material (Thy 5055 grave N16, x 166) for the current research. Two ^14^C dates from the burned bones in cist N16 date the burial to Period III (AAR-20592, 2966+/- 25 and AAR-20596, 2910 +/-25; both dates at 1-sigma confidence level) [12].

## **Early Nordic Bronze Age Zealand:**

### **Hvidegaard (Ke399, FF 020307-17)**

The grave of Hvidegaard lies north of Copenhagen and is one amongst a series of at least four mounds whose first recorded discovery dates from the summer of 1845. The mound was investigated by the National Museum of Denmark by C. J. Thomsen and assistants. They discovered that the cist (København Amt, Sokkelund Herred, Lyngby-Tårbæk Sogn, NM B 9220) had been carefully floored with pieces of flint on which had been placed a folded 1.75 m-long wool textile and a cowhide (placed with the hair facing inwards). This cowhide wrapped a pile of human cremains measuring 0.8 m [14].

The north side of the grave contained a sword blade in a leather sheath. The bottom part of the sheath was wrapped with a small leather band and was equipped with a leather loop under the mouth. This loop contained a broken leather band into which were inserted three small bronze double buttons. The wooden grip of the sword was only partially preserved. To the south of the sword lay a leather pouch. Fabricated from a single piece of the leather, the pouch was fashioned so as to be held closed by a round-headed bronze needle which interlaced leather eyelets from alternating sides of the pouch’s mouth. It seems to have been carried by means of a thin leather strap. The pouch’s contents were rather curious. It contained what may be considered a fairly standard Nordic Bronze Age elite male equipment: bronze tweezers, a strike-a-light in a leather case, a bronze knife (*Rahmengriffmesser*) in a sewn leather holder and a horse-headed razor (also wrapped in a thin leather cord) as well as an unusual collection of other items. These last include a falcon claw, two gastropod shells (one small and with a hole, one larger), a piece of amber, a small piece of ochre, a small wooden cube, a flint chip, some roots and bark and another small leather holder containing some pebbles and the jaw of a squirrel [14,8,15]. The artefacts typologically date the grave to Nordic Bronze Age Period III, a period corresponding to 1300-1100 BC [16–18]. For the present research, a single *pars petrosa* from Hvidegaard (NM B 9220, left side, from a young adult) was sampled for strontium isotope analysis.

### **Maglehøj (Ke 183, FF 010310-01)**

The site of Maglehøj in Frederiksborg was excavated in 1888 by Boye (Frederiksborg Amt, Lynge-Frederiksborg Herred, Oppy-Sundby Sogn, NM B 4092-95). The cremation grave found there was the central grave in the first of two mounds built upon the same site. The second phase of the mound contained the remains on inhumation burial as well as a clay pot while the third and final mound included the remains of a human cremation and several ceramic sherds. The central grave which concerns us here (Grave A) was covered in seaweed after completion [19]. Inside, the excavators found a paved floor on which lay woolen cloth wrapped around a pile of cremated human bones as well as a double button, a knife, a fibula and a bronze belt box. The artefacts typologically date the grave to Nordic Bronze Age Period III [16–18]. The contents of the Maglehøj belt box show striking similarities with the pouch from Hvidegaard. The belt box contained three small stones, a piece of sulfur, a rowan twig, charcoal, and a variety of diverse animal parts. These items were later identified by J. Steenstrup in 1888 as the pieces of two horse teeth, several weasel bones, the claw of a lynx, the bones of an unidentified mammal, the trachea of a bird, the vertebra of a snake (identified in 2008 to be that of an Aesculapian snake or *Zamenis longissimus*) and some cremated bones thought to be of human origin [20–22]. For the present research, a *pars petrosa* from Maglehøj (NM B 4092-95, right side, from an adult) was sampled for strontium isotope analysis.

## **Late Nordic Bronze Age Thisted County:**

### **Ginnerup (FF 110605-58)**

A single Late Nordic Bronze Age sample (from a *pars petrosa*) was also taken from the site of Ginnerup (Thy 5055 grave N30, x 147), which lay a few meters away from the central grave with the two Early Nordic Bronze Age children which was also sampled in the present study. The Late Nordic Bronze Age grave (THY 5055 grave N30) has been interpreted as a later addition to the mound and included a small wooden cist with capstone, possibly indicating the use of a wooden trough as the container of the cremated bones. The grave had been quite strongly disturbed by animal activity and, interestingly, the sample studied here was from human cremated material which had been found above the capstone. It is important to point out, however, that the displacement of the human material above the capstone was probably due to this same rodent activity [12]. Grave N30 was associated with a small bronze dagger (15 cm) in a wooden sheath, an unornamented double button and a small bronze spiral. Traces of some kind of packing material for the cremated remains were also recovered, which the excavators suggest may have been either textile or leather. Although this portion of the human remains from Ginnerup have not been anthropologically assessed, based on the double button, the human remains from grave N30 suggest a male individual who was likely interred in the Late Nordic Bronze Age Period IV. His inclusion within this study is intended as a later point of comparison for the other data.

### **Late Bronze Age sites from Stenildgård region in Vesthimmerland:**

## **VMÅ 2560 (FF 120814-75)**

It was in preparation for just such an expansion of the “Stenildhøj” housing development that rescue excavations by Vesthimmerlands Museum were conducted at the grave mound VMÅ 2560 (120814-75) in 2006. The mound contained a central grave from the Single Grave Culture, an inhumation from the Dagger Period, urns and cremation graves from the Bronze Age/Early Iron Age as well as pits with burned materials and two potential cult areas possibly connected to funerary rituals [23]. For the present research, we sampled material from two urns from the southern part of the grave mound for strontium isotope analysis (Urns A22 and A23).

The deposition of Urn A22 was carefully arranged. The conical urn was placed in a stone-packed cut and included a lid set in place with some type of sealant on top of which a flat stone had been placed. The urn contained charcoal and burned human bones as well as the shoulder blade and longbones of an avian. Osteological analyses suggest that the human bones represent those of an adult [23]. Additional grave goods included the tusk of a boar (which showed signs of having also been placed on the cremation pyre, as it was burned black) as well as one small bronze ring and a further bronze fragment. This urn likely dates from the Late Bronze Age (ibid). For the present research, we sampled an adult upper second molar (VMÅ 2560 x 133 Urn A22; KF1844) and a pars petrosa (VMÅ 2560 x 132 Urn A22; KF1842) from the human material as well as the boar tusk from the faunal material (VMÅ 2560 x 133 Urn A22; KF1843).

The second urn sampled from the south side of this mound was Urn A23, which was placed on a large stone within stone packing. Urn A23 differed from Urn 22 insofar as it was double conical in shape and had a thick lid with an interior lip (Falz). Like Urn A22, the lid of Urn A23 was closed with some kind of sealant, which the excavators note may possibly be birch tar [23]. Many of the bones within Urn A23 were blue-toned in colour, suggesting a low cremation temperature (ibid). Traces of green staining present on the bones are notable, probably as the result of bronze corrosion from the grave goods, as has been described from other contemporary contexts [24–27]. The urn contained fire-cracked flint, three unworked amber pieces, a finely-knapped arrowhead, a further broken fragment of another knapped object, a strike-a-light, a fossilized sea urchin, some bronze wire, a bronze needle, a bronze razor and a bronze awl. This urn likely dates from the Late Bronze Age (ibid). For the present research, we sampled a pars petrosa from this urn (VMÅ 2560 x 28 Urn A23; KF1840).

### **VMÅ 2542 (FF 120814-71)**

The second over-ploughed grave mound from which this study sampled material was atypical insofar as the central portion of the mound contained a possible grave surrounded by five post holes. Vesthimmerlands Museum’s 2006 excavation also uncovered the remainder of a Roman Iron Age grave discovered in the mound in 1921 in addition to a burned area and two urn graves from the Late Bronze Age. It is worth noting that a set of wheel tracks were also discovered to the south of the excavation area [28].

From this mound, we took a sample from a lower second molar from Urn A4 (VMÅ 2542 x13 Urn A4; KF1841), which was located on the southeast side of the mound. As was the case with the previous urns, VMA2542 Urn A4 was placed very carefully; in this instance, it was supported by four fist-sized stones. The urn was tall, narrow and double-conical in shape and had a small opening. Unfortunately, this urn was broken into many small fragments. The cremated material from the urn contained both bones and teeth. It may be important to note that the lack of wear on the surviving occlusal surfaces of the teeth may be indicative of the younger age of the deceased [28]. The urn also contained a bronze rivet with a round head ornamented with concentric circles as well as some seeds (x9). On the whole, the paucity of charcoal in the urn intimates that the bones may have been cleaned before they were placed inside. The excavators suggest a Late Bronze Age date for this context [28].

### **VMÅ 2883 (FF 120814-61 to 63)**

The most recent archaeological activities at Stenildhøj are the source for our final three samples. In 2018, the villa district in Stenildhøj in Aars was expanded. Before the construction, Vesthimmerlands Museum was given permission to investigate a range of Stone Age and Bronze Age funerary areas and settlement zones. In this way, the three overploughed grave mounds which yielded our last three samples (see below) were excavated as well as several other features and areas [29].

For the current research, three separate samples were taken from three separate mounds within the Stenildgårdbakken (VMÅ 2883) region. The first of these samples was a lower first or second premolar which was taken from Urn A267 (VMÅ 2883 x 136 Urn A267; KF 1838). Urn A267 was located on the periphery of mound A648 (120814-63), and counted as one of the mound’s four cremations. Urn A267 was tall and narrow with a faint double-conical shape and was placed near to a large stone on the eastern side of the mound. This lid of the urn included a central knob and was somewhat misshapen, which may have contributed to the fact that the lid had fallen into the urn at some point prior to its discovery. Typologically, the urn can be dated to the Late Bronze Age [29].

A further two samples were taken for the present study. One of these (a pars petrosa) came from Urn A349 (VMÅ 2883 x202; KF 1839). Urn A349 was one of three secondary urn graves within mound A650 (120814-61). This urn was placed on the northeastern side of the mound. Unfortunately, the top of the urn was lost due to plough damage. No further grave goods were apparent. However, typological analysis of the base of the urn allowed excavators to date it to the Late Bronze Age [29].

The final sample was taken from Urn A521, and was found in the periphery of grave mound A649 (120814-62). One of two secondary cremation graves within the mound, Urn A521 was placed in a little stone cist on the eastern side of mound A649. The cut for the deposition of the urn was deep and straight and was packed with flat stones which lined the urn on all sides as well as top and bottom. The bones contained within the urn where burned white. The urn can typologically be dated to the Late Bronze Age [29]. A *pars petrosa* was sampled from this urn (VMÅ 2883 x 174 Urn A521; KF 1837).

# **Bibliography:**

1. Vedsted J, Thomsen PO. Villerup Beretning. København; 1982.

2. Aner E, Kersten K. Die Funde der ältesten Bronzezeit des nordischen Kreises in Dänemark, Schleswig-Holstein und Niedersachsen: Thisted. Neumünster and Copenhagen: Nationalmuseet and Wachholtz; 2001.

3. Olsen A-LH, Bech J-H. Damsgård. En overpløjet høj fra ældre bronzealder per. III med stenkiste og ligbrændingsgrube. KUML. 1993;1993–1994: 158–198.

4. Olsen A-LH. Egshvile: A Bronze Age barrow with early urn graves from Thy. Journal of Danish Archaeology. 1992;9: 133–152.

5. Hornstrup KM, Olsen J, Heinemeier J, Thrane H, Bennike P. A new absolute danish bronze age chronology as based on radiocarbon dating of cremated bone samples from burials. Acta Archaeologica. 2012;83: 9–10. doi:https://doi.org/10.1111/j.1600-0390.2012.00513.x

6. Alexanderson V. Anthropological Analyses Egshvile. København; 1990.

7. Brøndsted J. Beretning Erslev. København; 1929.

8. Broholm HC. Danmarks Bronzealder. Copenhagen: Nyt Nordisk Forlag Arnold Busck; 1943.

9. Olsen A-LH. Nørhågaard. København; 1982.

10. Frei KM, Bergerbrant S, Sjögren K-G, Jørkov ML, Lynnerup N, Harvig L, et al. Mapping human mobility during the third and second millennia BC in present-day Denmark. PLOS ONE. 2019;14: e0219850. doi:10.1371/journal.pone.0219850

11. Andersen S. Katalog Antropologiske Analyser Nørhågaard. København;

12. Bech J-H, Roesgaard Hansen M. Thy 5055 Ginnerup: Beretning for udgravning af en overpløjet høj med centralgrav og to samhørende ligbrændningsgruber. København; 2014.

13. Skals I, Wiinblad T. Udgravning af bronzealdergrav fra Ginnerup optaget som præparat. Danmarks Grundforskningsfonds Center for Textilsforskning: Bevaringsafdelingen Nationalmuseet; 2013.

14. Thomsen CJ. Beretning Hvidegaard. 1845.

15. Aner E, Kersten K. Die Funde der älteren Bronzezeit des nordischen Kreises in Dänemark, Schleswig-Holstein und Niedersachsen. Frederiksborg und Københavns Amt. Neumünster and Copenhagen: Nationalmuseet and Karl Wacholtz Verlag; 1973.

16. Montelius O. Dating in the Bronze Age. Stockholm: K. Viterhets Historie och Antikvitetsakademien; 1986.

17. Jensen J. Danmarks Oldtid Bronzealder 2000-500 f. Kr. Copenhagen: Gyldendal; 2006.

18. Vandkilde H. From Stone to Bronze. The Metalwork of the Late Neolithic and Earliest Bronze Age in Denmark. Aarhus: Jysk Arkaeologisk Selskab/Aarhus University Press; 1996.

19. Boye V. Beretning Maglehøj. 1888.

20. Herbst CF. Hvidegaards Fundet. Annaler for Nordisk Oldkyndighed og Historie. 1848; 336–352.

21. Kristensen HV. Først danske fund af knogler fra æskulapsnog (*Zamenis longissimus*) i bronzealdergrav. Nordisk Herpetologisk Forening. 2008;3: 82–86.

22. Aaris-Sørensen K. Danmarks forhistoriske dyreverden. København: Glydendal; 1988.

23. Clemmensen B. Udgravningsberetning VMÅ 2560 Stenildhøjbakken. Slots og Kulturstyrelsen; 2007 pp. 1–28. Report No.: 120814–75, KUAS 2003-2122–1384.

24. Nørgaard HW. Portable XRF on Prehistoric Bronze Artifacts: Limitations and Use for the Detection of Bronze Age Metal Workshops. Open Archaeology. 2017;3: 101–122.

25. Oudbashi O, Emami SM, Ahmadi H, Davami P. Micro-stratigraphical investigation on corrosion layers in ancient Bronze artefacts by scanning electron microscopy energy dispersive spectrometry and optical microscopy. Heritage Science. 2013;1: 1–10.

26. Robbiola L, Blengino J-M, Fiaud C. Morphology and Mechanisms of formation of Natural Patinas on Archaeological Cu-Sn Alloys. Corrosion Science. 1998;40: 2083–2111.

27. Chase W. Chinese bronzes: casting, finishing, patination and corrosion. In: Scott D, Podnay J, Considine B, editors. Ancient and Historic Metals: Conservation and Scientific Research. Cincinnati, Ohio: J. Paul Getty Trust; 1994. pp. 85–118.

28. Clemmensen B. Udgravningsberetning VMÅ 2542 Stenildhøjbakken. Slots og Kulturstyrelsen; 2007. Report No.: 120814–71, KUAS 2003-2122–1199.

29. Nielsen SK. Udgravningsberetning VMÅ 2883 Stenildhøjbakken 2017, Års sogn, Års herred, Ålborg amt. Sted nr. 12.08.14-432. KUAS nr. 17/00070. Slots og Kulturstyrelsen; 2018 pp. 1–187. Report No.: 120814–423, KUAS No 17/00070.
