# Supplementary material for: Into the fire: Investigating the introduction of cremation to Nordic Bronze Age Denmark: A comparative study between different regions applying strontium isotope analyses and archaeological methods
Source: PLoS One. 2021 May 12;16(5):e0249476. doi: 10.1371/journal.pone.0249476 (PMC8115792; doi:10.1371/journal.pone.0249476)
Supplement: S2 Appendix — (DOCX) [file pone.0249476.s015.docx]

# **S2 Appendix. Osteological Analyses and Results**

## **Description of Methods**

In order to facilitate cross-comparison of osteological observations (especially MNI) between the different sites and study areas, the remains from the two cremations from Zealand (Hvidegaard and Maglehøj) were examined by Jørkov following the guidelines by Brickley and McKinley [1]. Sex was determined based on size and morphological features on cranial and post cranial elements following Buikstra and Ubelaker [2]. The effect of shrinkage and deformation due to the heat was considered when applying the methods. Age was determined based on epiphyseal fusion [3,4], dental development [5] and from signs of degenerative changes. The cremated remains were weighed and volume was established. In cases where the remains numbered less than 50 fragments, the remains were weighed and counted.

## **Results Osteological Analyses**

Our present analyses of Grave NM B 9220 (known as the Hvidegaard “shaman” grave) suggest the presence of the cremated remains of a minimum of two individuals; a young adult (18-25 years) and a small child (1-2 years). The total volume was 2175 ml and the weight was 793.8 g. For the adult remains alone, the volume was 2075 ml and the weight was 762.8 g. The total volume for the subadult cremated remains were 100 ml and the weight 31 g. Both individuals had skeletal elements represented from the majority of the skeleton, including elements from the skull and mandible (<25%), ribs (<25%), vertebrae (cervical, thoracic and lumbar) (25-50%) and pelvis (<25%), upper and lower extremities, and for the adult including hands and feet (carpals/tarsals, MC/MT and phalanges) (<25%). A single permanent tooth root (a lower left incisor) was present from the adult dentition. The child’s body was not represented by any elements from hands, feet or dentition.

The large size of some of the skeletal elements of the adult (a fragmented femoral caput and radial epiphysis) demonstrated characteristics consistent with male dimorphism. There were no signs of disease on the adult remains. A fragment of the left palate (maxilla) and vault from the child displayed porosities (*porotic hyperostosis*). The porosities in the palate were severe and could indicate that the child suffered from a vitamin deficiency either because of an infection or due to malnourishment. There were no signs of trauma (*ante* or *perimortem*) on any of the fragments.

The Hvidegaard material was exclusively thermally modified. All of the remains had been burned to the point of calcination with heat temperatures reaching more than 700 degrees; they appeared white-yellow or white-grey with varying brown discoloration from the soil. The fragmentation varied from high (crushed) to low (sizes bigger than 5cm). See Table 1. All of the bones appeared slightly shrunken, with small cracks, longitudinal- and thumb-nail fracture lines. Thumb-nail fractures were particularly evident in the long bone fragments. The cranial vault fragments of the adult and child also exhibited delamination (separation of inner and outer cortical table).

| **Site Name** | **Museum ID** | **Total MNI** | **MNI of adults** | **MNI of sub-adults** | **Nr of fragments** | **Volume (ml)** | **Weight**  **(g)** | **Colour** | **Degree of burning** | **Fragmentation** |
| --- | --- | --- | --- | --- | --- | --- | --- | --- | --- | --- |
| Hvidegaard | NM9220 | 2 | 1 | 1 | >1000 | 2175 | 793,8 | White-yellow-brown | >700 | Low-high |
| Maglehøj | NM B4092-95 | 1 | 1 |  | >1000 | 2035 | 958 | White-grey-black | 500->700 | Low-high |

**Table 1: Information table detailing osteological observations of cremated material from Hvidegaard and Maglehøj.**

The Maglehøj burial (NM4092-95) consisted of cremated remains of a single individual. Most of the cranial and postcranial skeleton was represented. There were elements from the skull and mandible (<25%), scapula and clavicle (<25%), ribs (<25%), vertebrae (cervical, thoracic, lumbar and sacrum) (25-50%) and pelvis (<25%), upper and lower extremities including hands and feet (carpals/tarsals, MC/MT and phalanges) (~25%). The roots of a lower canine and premolar was present from the dentition. The largest fragment was from the ilium and measured 5.7 x 3.2 cm. Green staining probably caused by contact with bronze objects was visible on several skeletal elements including thoracic and lumbar vertebrae, a fragment of a proximal radius and on a clavicle (medial end). A fragmented femoral caput and radial epiphysis provided size characteristics consistent with female dimorphism. The roots of the canine and premolar were closed, the femur head showed slight marginal lipping in the fovea, and marginal lipping could be observed on a lower vertebral body (an L5 or S1). The degenerative changes on the bone suggest that the individual was possibly 30 years or older. In addition, slight healed porosities could be observed in the palate of the maxilla and on a vault fragment (*porotic hyperostosis*).

The majority of the remains from Maglehøj appeared white-grey. Black charred areas were visible in the *diplöe* of cranial vault fragments and on a fragment of a proximal ulna, suggesting that calcination was not complete in those areas of the body. The degree of fragmentation varied, but was generally medium to high. See Table 1. All the long bones showed longitudinal thumb nail fractures. The cranial fragments were too small to identify signs of delamination.

The bones from all individuals analyzed from both sites appeared slightly warped. Warpage and delamination is associated with moisture in the bones and indicate that at least some (if not all) were covered with soft tissue or body fluids at the time of the fire. The varying degrees of fragmentation (of which a large portion were low) suggest that the remains were not deliberately damaged or crushed after burning, but fragmented due to heat and the burial environment.

# **Bibliography:**

1. Brickley M, McKinley JI. Guidelines for the Standards for Recording Human Remains. Southampton: Southampton University Press; 2004.

2. Buikstra JE, Ubelaker DH. Standards for Data Collection from Human Skeletal Remains. Arkansas Archaeological Survey; 1994.

3. Scheuer L, Black S. Developmental Juvenile Osteology. London: Academic Press; 2000.

4. Greulich WW, Pyle SI. Radiographic Atlas of Skeletal Development of the Hand and Wrist. 2nd ed. Redwood City: Stanford University Press; 1959.

5. AlQahtani SJ, Hector MP, Liversidge HM. Brief communication: The London atlas of human tooth development and eruption. Am J Phys Anthropol. 2010;142: 481–490. doi:10.1002/ajpa.21258
